# Time-dependent homeostatic mechanisms underlie brain-derived neurotrophic factor action on neural circuitry

**DOI:** 10.1038/s42003-023-05638-9

**Published:** 2023-12-18

**Authors:** Kate M. O’Neill, Erin D. Anderson, Shoutik Mukherjee, Srinivasa Gandu, Sara A. McEwan, Anton Omelchenko, Ana R. Rodriguez, Wolfgang Losert, David F. Meaney, Behtash Babadi, Bonnie L. Firestein

**Affiliations:** 1 Department of Cell Biology and Neuroscience, Rutgers University, Piscataway, NJ USA; 2 Biomedical Engineering Graduate Program, Rutgers University, Piscataway, NJ USA; 3 Department of Bioengineering, University of Pennsylvania, Philadelphia, PA USA; 4 Department of Electrical and Computer Engineering, University of Maryland, College Park, MD USA; 5 Cell and Developmental Biology Graduate Program, Rutgers University, Piscataway, NJ USA; 6 Neuroscience Graduate Program, Rutgers University, Piscataway, NJ USA; 7 Department of Physics, University of Maryland, College Park, MD USA; 8 Institute for Physical Science & Technology, University of Maryland, College Park, MD USA; 9 Department of Neurosurgery, University of Pennsylvania, Philadelphia, PA USA; 10 Present Address: Institute for Physical Science & Technology, University of Maryland, College Park, MD USA

**Keywords:** Neural circuits, Cellular neuroscience, Network models

## Abstract

Plasticity and homeostatic mechanisms allow neural networks to maintain proper function while responding to physiological challenges. Despite previous work investigating morphological and synaptic effects of brain-derived neurotrophic factor (BDNF), the most prevalent growth factor in the central nervous system, how exposure to BDNF manifests at the network level remains unknown. Here we report that BDNF treatment affects rodent hippocampal network dynamics during development and recovery from glutamate-induced excitotoxicity in culture. Importantly, these effects are not obvious when traditional activity metrics are used, so we delve more deeply into network organization, functional analyses, and in silico simulations. We demonstrate that BDNF partially restores homeostasis by promoting recovery of weak and medium connections after injury. Imaging and computational analyses suggest these effects are caused by changes to inhibitory neurons and connections. From our in silico simulations, we find that BDNF remodels the network by indirectly strengthening weak excitatory synapses after injury. Ultimately, our findings may explain the difficulties encountered in preclinical and clinical trials with BDNF and also offer information for future trials to consider.

## Abstract (web summary)

A study uses MEA analysis, computation, and simulations to demonstrate how BDNF treatment after excitotoxic injury remodels hippocampal networks by promoting inhibitory neuron survival while indirectly strengthening weak excitatory synapses.

## Introduction

Biological systems tend toward maintaining homeostasis after being perturbed^1,2^, which is especially true for neuronal networks^3^. Indeed, permanently disrupted homeostasis in developing neurons may lead to neuropsychiatric disorders (reviewed in ref. ^4^). Perhaps the most impressive aspect of brain development is the seemingly paradoxical relationship between rewiring activity and circuit stability, given how developing neuronal networks maintain appropriate function in the midst of extensive rewiring (reviewed in ref. ^5^). Moreover, homeostasis can occur at multiple levels, potentially resulting in changes to individual neurons while maintaining similar activity at the network level (reviewed in ref. ^6^).

Each of these physiological properties of homeostasis across scales within the brain is challenged when the same stable neuronal networks are injured. While the type and severity of injury are most predictive of functional recovery, neuronal networks can display a great amount of resilience^7^. In particular, the ability of developing networks to recover after injury is likely due to increased plasticity since both structural and functional plasticity increase within and near the site of injury in vivo^8^. Similar to developmental changes, an injured network must properly rewire itself during the recovery phase, and the correct connections must be remade^9^. Although developing networks and injured networks demonstrate similar changes in protein expression^10^, the corresponding properties of network-level function in neither developing nor injured networks have been investigated in depth. Our broad goal is to study the principles of network homeostasis in neural circuits by examining if the intrinsic molecular mechanisms of rewiring are capable of shifting the homeostatic set point.

Several molecules regulate homeostatic plasticity, and the first secreted factor to be identified was brain-derived neurotrophic factor (BDNF). BDNF plays an important role in synaptic scaling by regulating the balance of excitation to inhibition (E/I) at cortical synapses^11^. Application of exogenous BDNF prevents alterations to miniature excitatory postsynaptic currents after activity is blocked^11^, suggesting a role for activity-dependent BDNF release in regulating synaptic scaling. BDNF is also important for inhibitory synapse development^12^ with activity-dependent release of BDNF being critical for synaptic scaling of inhibitory neurons^13^. Despite evidence that BDNF is the most prevalent growth factor in the central nervous system and can regulate multiple features of connectivity at the single-neuron scale, BDNF-promoted homeostasis has not been studied at the network level. We seek to fill this gap in our knowledge and determine how BDNF acts upon circuits. BDNF may help networks restore their intrinsic stability, and thus, BDNF treatment may help to reassemble an injured circuit. However, BDNF may mediate changes to network connectivity that impair the rebuilding process, which may explain why BDNF has not shown promising results in clinical trials^14^. Past work indirectly suggests that both effects are possible, yet there is no systematic study of networks at different scales that answers how BDNF affects circuits directly.

Here, we use microelectrode array (MEA) technology to record spontaneous network activity from primary rat hippocampal networks during development and after chemical injury. From our data obtained at high temporal resolution, we build upon our previous studies^15–18^ and use activity parameters for individual electrodes (representing small groups of neurons) to measure the functional network-level properties before and after remodeling the network with BDNF treatment. To obtain a directional network characterization of the MEA data, we use Granger causality (GC) analysis, which statistically tests whether a source time series significantly improves the forecasting of a target time series. GC analysis has frequently been used for continuous neuroimaging data as an established functional network characterization methodology^19–23^; here, we employ recent extensions that enable its application to binary event data^24,25^ and, in particular, model the sparse interactions that occur in neuronal networks^25^. We additionally analyzed higher-order synchronous activity (i.e., simultaneous activity shared across more than two electrodes) to complement the pairwise analysis offered by GC. Finally, to inspect the circuit-level mechanisms that could underlie the changes in functional networks, we developed a computational model of neuronal network activity that examines specific mechanisms of how the exogenous application of BDNF affects remodeling in neuronal networks during recovery from injury.

Since BDNF is a well-known regulator of neuronal morphology and synaptic activity^11,12,26–30^, we investigated whether BDNF application alters the activity of developing networks. We treated hippocampal networks with 25 or 50 ng/ml BDNF, two concentrations that affect the morphology of the dendritic tree in neurons in our networks^27,29^. Only the higher concentration decreases TrkB phosphorylation, an effect that lasts for 7 days after treatment ends and demonstrates that the two concentrations differ in their action. Both concentrations of BDNF alter the local efficiency and synchronization of developing networks, although with distinct effects and on different timescales.

Given the involvement of BDNF in neuroprotection when applied prior to injury^31,32^, we then investigated whether treatment with 50 ng/ml BDNF rescues injury-related changes caused by glutamate-induced excitotoxicity. We found that BDNF exacerbates injury-induced decreases in burstlet rate and local efficiency while preventing injury-induced decreases to weak and medium synchronized connections. Our results suggest that BDNF acts on network activity in a manner that maintains much of the synchrony between electrodes while allowing the overall firing rate to decrease. Moreover, network-level properties inferred from higher-order synchrony and GC network analyses corroborate these findings by revealing mixed effects of BDNF treatment in injured networks. Finally, our simulated in silico networks suggest that, although BDNF promotes inhibitory neuron survival following injury, it remodels the network by indirectly affecting excitatory synapses. Taken together, our results demonstrate complex roles for BDNF in the regulation of E/I balance during development and after injury.

## Results

### Sustained BDNF-promoted decreases in phosphorylated TrkB expression in hippocampal networks depend on BDNF concentration

**Fig. 1:**
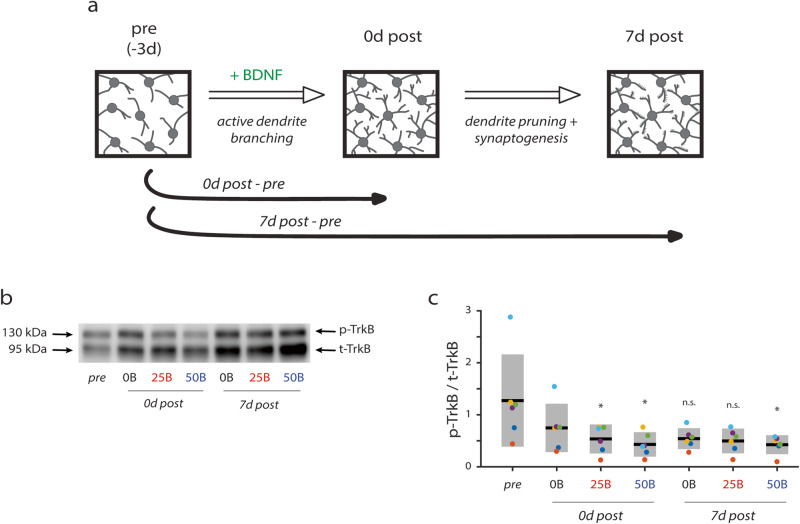
Changes in ratio of phosphorylated (p-TrkB) to total TrkB (t-TrkB) in cultured hippocampal neurons in response to BDNF treatment. **a** Schematic illustrating timepoints for data acquisition. Pre timepoint is DIV 7. **b** Representative blot showing changes in p-TrkB (~140 kDa) and t-TrkB (~90 kDa) over time. **c** Quantification of p-TrkB/t-TrkB over time. Treatment with 25 ng/ml BDNF (25B) or 50 ng/ml (50B) causes decreases in p-TrkB/t-TrkB at 0d post-treatment compared to pre-treatment levels, but only treatment with 50B causes decreases at 7d post-treatment compared to pre-treatment levels (*p < 0.05). 0B = no BDNF; 25B = 25 ng/ml BDNF; 50B = 50 ng/ml BDNF. *p* values calculated by repeated measures ANOVA, and * indicates significant differences compared to pre-treatment levels. Solid black lines indicate the mean, and gray boxes indicate 95% CIs. Differently colored datapoints indicate individual trials. *N* = 6 independent experiments.

The concentration of BDNF used to treat neuronal networks is important since it influences receptor activation and downstream signaling. Similar to our previous work, we treated hippocampal networks with 25 or 50 ng/ml BDNF^27,29^. The lower concentration is thought to target TrkB specifically, whereas the higher concentration likely also activates TrkA and TrkC^27,29,33^. To determine whether these two concentrations distinctly affect the TrkB receptor, we treated networks with 0, 25, or 50 ng/ml BDNF (abbreviated 0B, 25B, and 50B, respectively) from day in vitro (DIV) 7 to 10. Importantly, this treatment window matches our previous work that quantified BDNF-mediated effects on the dendritic arbor of hippocampal neurons^27,29^. We then performed Western blot analysis and compared the ratio of phosphorylated TrkB to total TrkB (p-TrkB/t-TrkB) before treatment (“pre”), immediately after treatment (“0d post”), and one week after treatment ended (“7d post”; developmental timepoints indicated in Fig. 1a; all blots shown in Supplementary Fig. 1) to allow for assessment of short- and long-term effects of BDNF treatment. Repeated measures analysis of variance (RM ANOVA) reveals that timepoint has a significant effect on the p-TrkB/t-TrkB ratio (*F* = 20.5, *p* < 0.001; Supplementary Table 1). We then performed multiple comparisons analysis (i) based on BDNF concentration for each timepoint separately and (ii) based on the timepoint for each BDNF concentration separately. These analyses reveal that treatment with either 25B or 50B causes significant decreases in p-TrkB/t-TrkB ratio immediately after treatment (0d post); however, this decrease in TrkB phosphorylation is only sustained by the 50B treatment through 7 days after treatment ended (7d post; Fig. 1b, c). Our results suggest that these two concentrations of BDNF exert distinct effects on hippocampal networks but only when timepoint is taken into account.

### BDNF treatment causes time-dependent changes to local network efficiency in a concentration-dependent manner

**Fig. 2:**
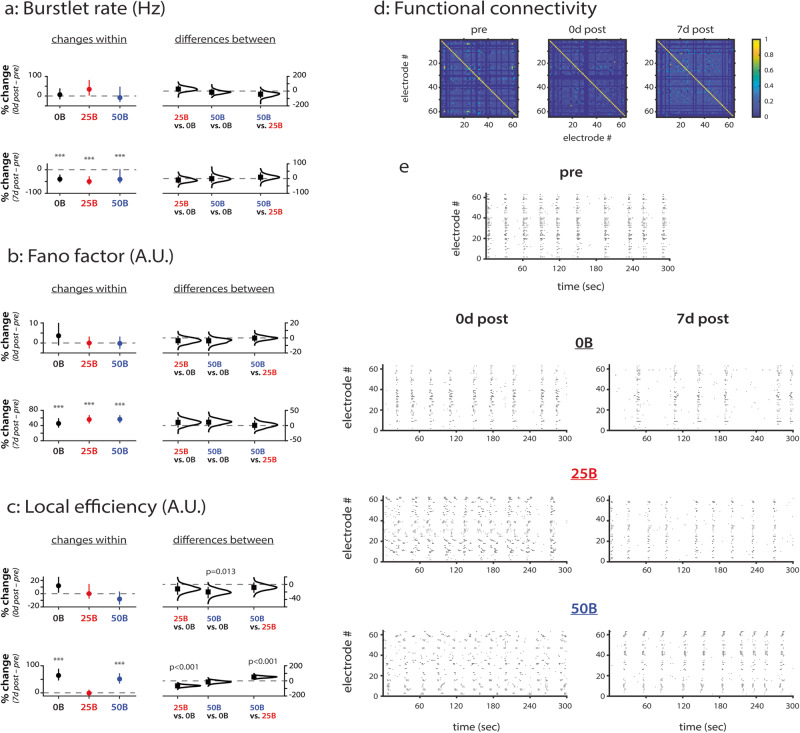
BDNF treatment alters local efficiency but not network activity. **a** Changes in burstlet rate (Hz). *e* = 158 for 0B; *e* = 184 for 25B; *e* = 180 for 50B. **b** Changes in Fano factor (A.U.). e=332 for 0B; *e* = 506 for 25B; *e* = 452 for 50B. **c** Changes in local efficiency (A.U.). *e* = 356 for 0B; *e* = 453 for 25B; *e* = 387 for 50B. **d** Functional connectivity matrices used for calculating local efficiency. Shown are representative matrices at each timepoint for the control condition. Colorbar represents the strength of cross-correlation. **e** Representative raster plots for each condition. 0B = no BDNF; 25B = 25 ng/ml BDNF; 50B = 50 ng/ml BDNF. Data in **a**–**c** from *N* = 3 independent experiments. For **a**–**c**: *y*-axis indicates percent change from baseline (pre-treatment). Top row compares pre-treatment to 0d post-treatment, and the bottom row compares pre-treatment to 7d post-treatment. Plots on the left indicate means with 95% CIs, and *p* values calculated by RM ANOVA followed by Tukey–Kramer multiple comparisons test, where *p < 0.05, ***p < 0.001 indicate significant differences between timepoints within the same condition. Distributions on the right with means (black squares) and 95% CIs (vertical lines) show comparisons between conditions and were calculated via estimation statistics with *p* values calculated directly from CIs. *N* indicates the number of experiments, and *e* indicates number of electrodes.

We next sought to investigate whether BDNF treatment alters network activity during hippocampal network development. To this end, we recorded spontaneous activity using microelectrode arrays at the same developmental timepoints (experimental timeline shown in Supplementary Fig. 2a) to assess short- and long-term effects of BDNF treatment on network dynamics (Fig. 2; all data points shown in Supplementary Fig. 3a–c). To achieve the most complete analysis of our rich MEA data, we used RM ANOVA and multiple comparisons to analyze changes within BDNF treatment conditions over time (“changes within” on left sides of plots; significance represented by asterisks) followed by estimation statistics^34^ to quantify whether the changes over time differ across treatment conditions (“differences between” on right sides of plots; significance represented by *p* values). It is important to note that, for MEA data, we are only interested in changes over time because each in vitro network develops uniquely and, thus, requires normalization. Therefore, all MEA data are expressed as percent change (% change). We offer additional explanations of statistics used in the “Methods” section (subsections: “Data representation” and “Statistics and reproducibility”).

First, when using RM ANOVA analysis, we find that burstlet rate (a measure of organized activity) is significantly affected by timepoint (*F* = 21.7, *p* < 0.001; Supplementary Table 1). Multiple comparisons testing further reveals (i) that burstlet rate does not change significantly for any condition immediately after treatment ends compared to pre-treatment (Fig. 2a, plots on left in top panel, 0d post vs. pre) and (ii) that, regardless of condition, burstlet rate significantly decreases by ~40% at seven days after treatment ends compared to pre-treatment (Fig. 2a, plots on left in bottom panel, 7d post vs. pre). Moreover, using estimation statistics, we find that none of the treatment conditions significantly differ from one another (Fig. 2a, distributions on right in top and bottom panels, 0d post vs. pre and 7d post vs. pre).

Since we did not observe differences between BDNF treatment conditions in organized network activity (i.e., burstlet rate), we next investigated whether spiking variability—as measured by the Fano factor—is changed by BDNF treatment or during development. RM ANOVA analysis reveals that timepoint significantly affects spiking variability as measured by the Fano factor (*F* = 209, *p* < 0.001; Supplementary Table 1). After multiple comparisons testing, we find that spiking variability does not change at 0d post-treatment (DIV 10) compared to baseline for any condition (Fig. 2b, plots on left in top panel, 0d post vs. pre) but that it significantly increases at 7d post-treatment (DIV 17) compared to baseline for all conditions (Fig. 2b, plots on left in bottom panel, 7d post vs. pre; *p* < 0.001 for 0B, 25B, and 50B). Spiking variability therefore appears to be a robust metric related to network maturity since, compared to baseline, the Fano factor increases by 46% in control networks at 7d post-treatment (and by 56% and 57% in 25B and 50B networks, respectively). Regardless of other changes that result from BDNF treatment, such as TrkB phosphorylation levels, our results suggest that hippocampal networks maintain homeostasis by following a specific maturation program of gradually increasing spiking variability.

We next examined whether network age or BDNF treatment alters the local efficiency of hippocampal networks (Fig. 2c). We calculate local efficiency as previously reported^17^, where we use cross-correlation of binned spiking activity to generate functional connectivity matrices (Fig. 2d); representative matrices are shown for the control condition at all timepoints. RM ANOVA reveals that BDNF treatment (*F* = 10.1, *p* < 0.001), timepoint (*F* = 47.6, *p* < 0.001), and the interaction of BDNF treatment and timepoint (*F* = 14.1, *p* < 0.001) significantly affect local efficiency (Supplementary Table 1). Multiple comparison testing reveals additional details. Regardless of condition, all networks demonstrate the same local efficiency immediately after treatment compared to baseline (Fig. 2c, plots on left in top panel, 0d post vs. pre). However, estimation statistics reveal that the 50B treatment significantly decreases local efficiency (by 20%) at this timepoint compared to control networks (Fig. 2c, *p* = 0.013 for 50B vs. 0B distribution on right in top panel, 0d post vs. pre). Despite this short-term difference between the 0B and 50B conditions, no differences in local efficiency are observed between them at 7 days after treatment: both show significantly increased local efficiency (64% and 51% for 0B and 50B, respectively) compared to baseline (Fig. 2c, plots on left in bottom panel, 7d post vs. pre; *p* = <0.001 for 0B and 50B). Treatment with 25B has no effect on local efficiency (assessed by RM ANOVA), with estimation statistics revealing that this lack of effect for 25B is significantly different from the changes observed in 0B and 50B networks (Fig. 2c, *p* < 0.001 for 25B vs. 0B and for 50B vs. 25B distributions on right in bottom panel, 7d post vs. pre). We also quantified global efficiency but found no differences over time or between treatment conditions (data not shown).

Taken together, our results suggest that (i) network age is the most consistent variable that changes network activity, (ii) hippocampal networks are relatively resilient to treatment-induced network-level changes during development (e.g., burstlet rate and Fano factor), and (iii) any effects exerted by BDNF (e.g., on local efficiency) are concentration-dependent (Fig. 2e).

### BDNF treatment alters synchronization in a concentration-dependent manner

**Fig. 3:**
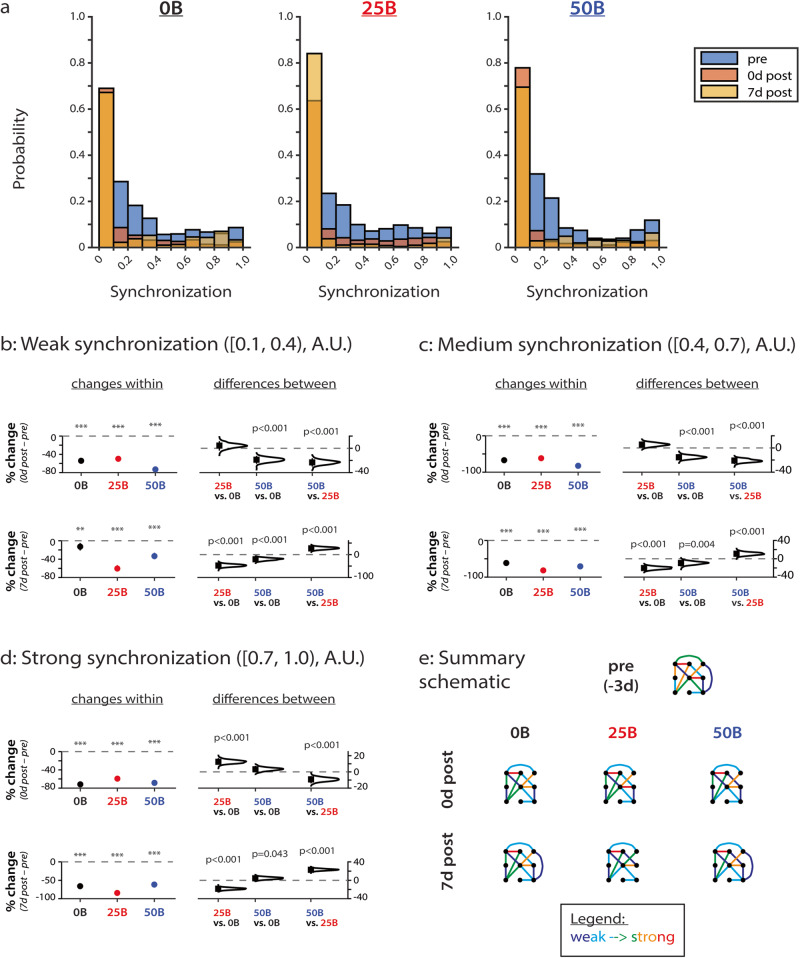
BDNF treatment distinctly alters connection strength in a concentration-dependent manner. **a** Distribution of synchronization strengths for cultures treated with vehicle (0B; left), 25 ng/ml BDNF (25B; middle), and 50 ng/ml BDNF (50B; right) over time. **b** Changes in connections with initially weak synchronization (values of [0.1,0.4)). *e* = 1720 for 0B; *e* = 1454 for 25B; *e* = 2002 for 50B. **c** Changes in connections with initially medium synchronization (values of [0.4,0.7)). *e* = 556 for 0B; *e* = 700 for 25B; *e* = 482 for 50B. **d** Changes in connections with initially strong synchronization (values of [0.7,1.0)). *e* = 620 for 0B; *e* = 654 for 25B; *e* = 760 for 50B. **e** Summary schematic of synchronization changes during development with BDNF treatment. Colors correspond to synchronization values, ranging from weak (dark blue) to strong (red). Data in **a**–**d** from *N* = 3 independent experiments. For **b**–**d**: *y*-axis indicates percent change from baseline (pre-treatment). Top row compares pre-treatment to 0d post-treatment, and bottom row compares pre-treatment to 7d post-treatment. Plots on the left indicate means with 95% CIs, and *p* values calculated by RM ANOVA followed by Tukey–Kramer multiple comparisons test, where ***p < 0.001 indicates significant differences between timepoints within the same condition. Distributions on the right with means (black squares) and 95% CIs (vertical lines) show comparisons between conditions and were calculated via estimation statistics with *p* values calculated directly from CIs. *N* indicates the number of experiments, and *e* indicates the number of electrodes.

As in our previous work^15,16^, we calculated the synchrony of firing (SF) between electrodes as a metric for synchronization (Fig. 3; all data points shown in Supplementary Fig. 3d–f), and it indicates how often electrodes show bursting behavior at the same time (simultaneous organized activity). The distributions of synchronization for all conditions are skewed towards weaker connections, quantitatively indicating that our in vitro networks do not demonstrate over-synchronization during development (Fig. 3a). We separated synchronization into three categories: weak (with values between [0.1, 0.4); Fig. 3b), medium (with values between [0.4, 0.7); Fig. 3c), and strong (with values between [0.7, 1.0); Fig. 3d), similar to what we have previously reported^15,16^. Categorization allows us to reveal how connections with specific initial strengths change over time and with treatment. A value of 0 indicates no synchronization between a pair of electrodes, and thus, we do not analyze initial connection strengths below 0.1 to prevent the inclusion of inflated positive changes after normalization^15,16^. We again use the same strategy of RM ANOVA and multiple comparisons to analyze changes over time within conditions and estimation statistics^34^ to determine whether changes over time are different across conditions when analyzing the data presented in Fig. 3.

All categories of synchronization significantly decrease in strength over time, but BDNF treatment changes the timing and extent of these decreases. RM ANOVA reveals that, for all categories of synchronization (weak, medium, and strong), BDNF concentration (*F* = 25.7, *F* = 14.4, *F* = 9.91, respectively; all *p* < 0.001), timepoint (*F* = 435, *F* = 1790, *F* = 3210, respectively; all *p* < 0.001), and the interaction between BDNF concentration and timepoint (*F* = 40.9, *F* = 31.8, *F* = 58.7, respectively; all *p* < 0.001) significantly affect each category of synchronization (Supplementary Table 1).

Weak and medium strength connections demonstrate similar changes as revealed by multiple comparisons testing. Treatment with 50B decreases synchronization (73% and 83%, respectively; *p* < 0.001 for both) to a greater degree than does treatment with 0B (54% and 67%, respectively; *p* < 0.001 for both) or 25B (50% and 62%, respectively; *p* < 0.001 for both) immediately after treatment compared to baseline (Fig. 3b, c, plots on left and distributions on right in top panel, 0d post vs. pre; p < 0.001 for 50B vs. 0B and for 50B vs. 25B). At 7d post-treatment compared to baseline, treatment with 25B causes greater decreases in weak and medium synchronizations (60% and 82%, respectively; *p* < 0.001 for both) compared to control (13% [*p* = 0.001] and 61% [*p* < 0.001], respectively) and 50B (33% and 71%, respectively; *p* < 0.001 for both) networks (Fig. 3b, c, plots on left and distributions on right in the bottom panel, 7d post vs. pre; *p* < 0.001 for 25B vs. 0B and for 50B vs. 25B).

Strong connections demonstrate other changes. At 0d post-treatment compared to baseline, estimation statistics reveal that treatment with 25B partially attenuates the decrease in synchronization observed in control (by 12%) and 50B (by 9.2%) networks (Fig. 3d, *p* < 0.001 for 25B vs. 0B and for 50B vs. 25B distributions on right in top panel, 0d post vs. pre). In contrast, at 7d post-treatment compared to baseline, estimation statistics show that networks treated with 25B demonstrate a significantly larger decrease in strong synchronization compared to control (by 18%) and 50B (by 23%) networks (Fig. 3d, *p* < 0.001 for 25B vs. 0B and 50B vs. 25B distributions on right in bottom panel, 7d post vs. pre). Moreover, treatment with 50B partially attenuates the decrease in synchronization (by 3.3%) observed in control networks (Fig. 3d, p = 0.043 for 50B vs. 0B distribution on the right in the bottom panel, 7d post vs. pre).

Thus, in the context of our study (Fig. 3e), BDNF treatment and network age significantly affect all categories of synchronization. Moreover, treatment with 25B attenuates changes to strong connections in the short-term (immediately after treatment compared to baseline), and treatment with 50B attenuates changes to strong connections in the long-term (at 7d after treatment compared to baseline).

### Functional network-level analyses reveal concentration-dependent effects of BDNF on developing networks

**Fig. 4:**
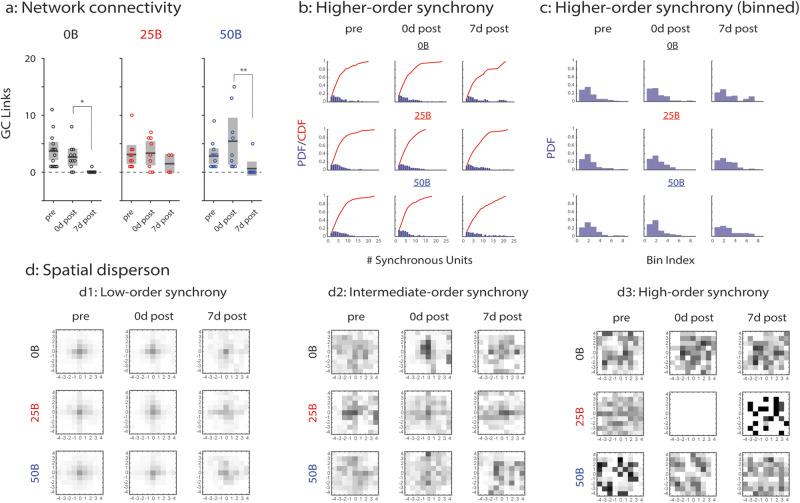
Granger causality (GC) and synchrony analyses of BDNF treated networks. **a** All GC links in control (0B; left), 25 ng/ml BDNF (25B; middle), and 50 ng/ml BDNF (50B; right) treated networks. *p < 0.01 and **p < 0.001; *represents significant differences between timepoints of the same condition based on two-sided Wilcoxon’s rank sum test. Solid black lines indicate the mean, and gray boxes indicate 95% CIs. **b** Higher-order synchrony frequency distributions shown as PDFs and CDFs for 0B (top row), 25B (middle row), and 50B (bottom row) treated networks. **c** Binned higher-order synchrony frequency distribution shown as PDFs for 0B (top row), 25B (middle row), and 50B (bottom row) treated networks. The distribution is weakly bimodal in control networks 7d post (Tokeshi’s test of bimodality; see Table 1). **d** Spatial dispersion of synchronized electrodes grouped by low-order synchrony (d1), intermediate-order synchrony (d2), and high-order synchrony (d3). Statistically significant *p* values for data in **d** included in Supplementary Material and were determined by two-sample KS tests. Top row: control networks; middle row: 25B treated networks; bottom row: 50B treated networks. Data from *N* = 7 independent experiments. 0B: *n* = 19 for pre; *n* = 15 for 0d post; *n* = 12 for 7d post. 25B: *n* = 21 for pre; *n* = 17 for 0d post; *n* = 14 for 7d post. 50B: *n* = 17 for pre; *n* = 14 for 0d post; *n* = 12 for 7d post. *N* indicates the number of experiments, and *n* indicates the number of datapoints (MEA networks).

BDNF-mediated changes in activity patterns and network efficiency demonstrate that BDNF treatment can alter network properties but offer no clear evidence on whether BDNF affects information flow through hippocampal networks. To address this knowledge gap, we adapted Granger causality (GC) analysis^25^ to infer directed networks between MEA electrodes based on bursting (organized) activity and examined how network connectivity changes in developing networks over time and with BDNF treatment (Fig. 4a). In our application, GC analysis determines if predictions of the activity of one electrode are significantly improved by the recent activity history of another electrode; analyzing all electrode pairs in this manner yields a directed functional network, where connectivity is summarized by the number of detected GC links.

We find that control networks (0B) and networks treated with 50B behave similarly, showing significant changes in the number of GC links at 7 days after treatment (7d post) compared to immediately after treatment (0d post; 0d post vs. 7d post, *p* < 0.01 for 0B and *p* < 0.001 for 50B) but no changes between baseline (“pre”) and 0d post-treatment or between baseline and 7d post-treatment. The number of GC links for networks treated with 25B does not change over time, indicating that treatment with 25B prevents long-term decreases in Granger causal links, hence maintaining network connectivity. The concentration-dependent effects of BDNF on GC network connectivity mirror the effects on weak, medium, and strong synchronization (Fig. 3b–d).

While GC analysis describes pairwise interactions as networks develop, it is unable to characterize the higher-order interactions between groups of three or more electrodes. Hence, we adapted a recently developed model of higher-order synchronized spiking^35,36^ to further assess the effects of BDNF on bursting (organized) activity. The model tests if all *r*th-order events (simultaneous burstlets across exactly *r* electrodes) occurred at a significantly high rate. We plotted frequency distributions of detected orders of synchrony for all timepoints and all treatment conditions; both cumulative distribution functions (CDFs) and probability distribution functions (PDFs) are included (Fig. 4b).

No significant changes in distribution are observed between baseline (“pre”) and 0d post-treatment for any condition (Fig. 4b, left and middle columns). However, a difference emerges between the control and BDNF treatment conditions at 7d post-treatment: the frequency distribution of higher-order synchrony in control networks appears bimodal, unlike in networks treated with 25B or 50B (Fig. 4b, right column and Table 1). We quantified the change in distributions by first binning the PDFs in Fig. 4b to generate the histograms (Fig. 4c). After applying Tokeshi’s test for bimodality^37^ (as described in “Methods” subsection “Tokeshi’s test of bimodality”), we find the distribution of detected orders of synchrony in control networks at 7d post-treatment to be weakly bimodal, whereas the distributions for networks treated with 25B and 50B are unimodal (Table 1). This finding indicates that BDNF treatment, regardless of concentration, attenuates the tendency of developing networks to become either globally or locally synchronized, instead promoting heterogeneous higher-order synchrony. These results, combined with GC findings, suggest an inhibitory network-wide effect of BDNF that varies by concentration.

**Table 1:** Tokeshi’s test of bimodality.

	${P}_{{\rm {c}}}$	${P}_{\rm {{l}}}$	${P}_{{\rm {r}}}$	$\rho (3)$
Uniform	>0.05	–	–	–
Unimodal	<0.05	<0.05	≥0.9	–
	<0.05	≥0.9	<0.05	–
Strongly bimodal	<0.05	<0.05	<0.05	–
Bimodal	<0.05	<0.25	<0.25	–
Weakly bimodal	<0.05	<0.5	<0.5	–
	<0.05	≥0.5	<0.05	<0.1
	<0.05	<0.05	≥0.5	<0.1

Next, to characterize the spatial extent of BDNF-mediated network-level effects, we analyzed the relationship between degree of spatial localization of synchronized units (electrodes) and orders of synchrony (i.e., the size of a group of synchronized electrodes), both across time and across conditions. We computed the distances of synchronized electrodes to their centroids and compiled histograms of these relative distances, thus, individually characterizing low-order synchronous electrodes (of order 2–8; Fig. 4d1), intermediate-order synchronous electrodes (of order 9–15; Fig. 4d2), and high-order synchronous electrodes (of order 16 or greater; Fig. 4d3). Distributions were compared using two-sample Kolmogorov–Smirnov (KS) tests.

The distributions of low-order synchronous electrodes were first compared at baseline and immediately after treatment (Fig. 4d1, left vs. middle column). For networks treated with 50B, the distribution becomes significantly more concentrated immediately after treatment compared to baseline (*p* = 0.025). At 7d post-treatment compared to 0d post-treatment (Fig. 4d1, middle vs. right column), the distribution is significantly more concentrated for control networks but not for networks treated with 25B or 50B (0B: *p* = 0.0186; 25B: *p* = 0.270; 50B: *p* = 0.140). Hence, BDNF treatment at either concentration promotes the dispersion of low-order connections at 7d after treatment.

Intermediate-order synchronous electrodes (Fig. 4d2, left column) are more dispersed than low-order synchronous electrodes (Fig. 4d1, left column) at baseline in control and networks treated with 50B (0B: *p* < 0.001; 50B: *p* < 0.001), while in networks treated with 25B, intermediate-order synchronous electrodes are similarly spread as low-order synchronous electrodes (*p* = 0.208). At 0d post-treatment (Fig. 4d2, middle column), the distribution of intermediate-order synchronous electrodes becomes more concentrated in all conditions (0B: *p* < 0.001; 25B: *p* = 0.044; 50B: *p* = 0.011). At 7d post-treatment (Fig. 4d2, right column), the distribution becomes significantly more dispersed than at 0d post-treatment in control networks (*p* = 0.013) and significantly less dispersed in networks treated with 25B or 50B over the same period (25B: *p* = 0.001; 50B: *p* < 0.001). These findings suggest that, at 7d post-treatment, BDNF treatment at either concentration inhibits the dispersion of intermediate-order connections.

High-order synchronous electrodes (Fig. 4d3, left column), like intermediate-order synchronous electrodes, are more dispersed than low-order synchronous electrodes (Fig. 4d1, left column) at baseline (*p* < 0.001 for each treatment condition). However, in neither control networks (first row) nor with 50B treatment (bottom row) does the spread of high-order electrodes change between baseline and 0d post-treatment (0B: *p* = 0.101; 50B: *p* = 0.122. 0d post vs. pre) or between 0d post- and 7d post-treatment (0B: *p* = 0.698; 50B: *p* = 0.168; 7d post vs. 0d post). Treatment with 25B (middle row) causes an absence of high-order synchronous electrodes at 0d post-treatment, and only four groups of such electrodes were detected at 7d post-treatment. Hence, our results suggest that high-order synchrony during network development is disrupted by low-concentration BDNF treatment (25B) but not by high-concentration treatment (50B).

Summarily, our analyses of functionally directed networks and higher-order synchrony suggest that, while concentration-dependent, the network-level effect of BDNF treatment on developing networks is functionally inhibitory. Low-concentration BDNF treatment maintains network connectivity but inhibits high-order synchrony in developing networks, while high-concentration BDNF treatment maintains high-order synchrony but inhibits network connectivity.

### Treatment with 50 ng/ml BDNF preferentially protects inhibitory over excitatory neurons from glutamate-induced excitotoxicity

**Fig. 5:**
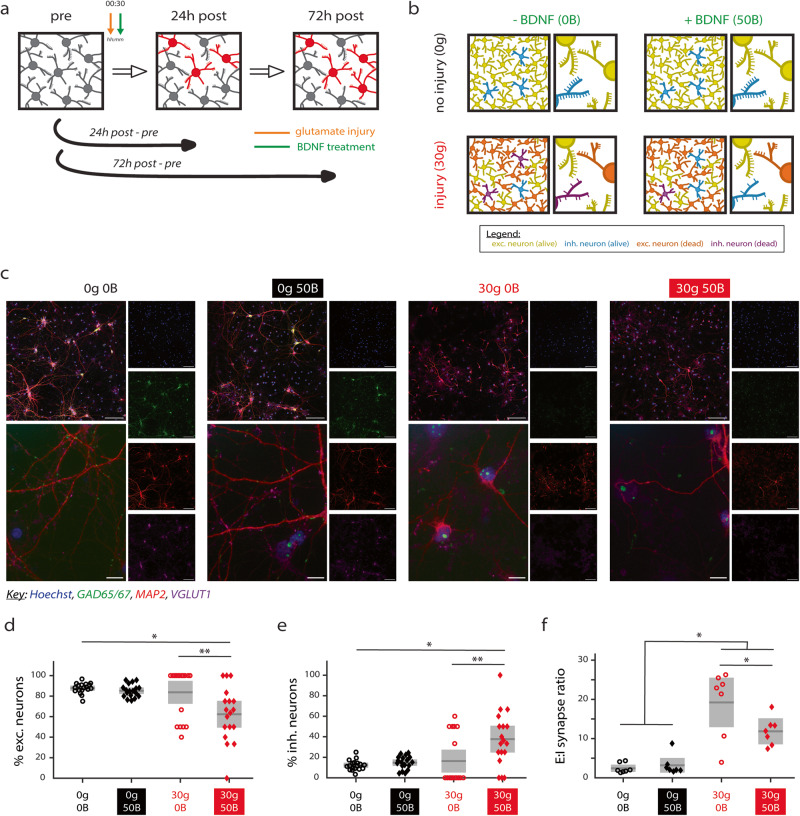
Glutamate-induced excitotoxicity and BDNF treatment alter excitatory/inhibitory (E/I) neuron and synapse balance. **a** Schematic illustrating timepoints for data acquisition; 00:30 indicates 30 min glutamate exposure (orange arrow) before BDNF treatment (green arrow). BDNF treatment persisted for 72 h. Pre timepoint is DIV 14. **b** Summary schematic of findings, illustrating that BDNF treatment ameliorates some aspects of injury with glutamate. **c** Representative images for E/I cell death studies (square tiles, merged and separate channels; scale bar indicates 100 μm) and E/I synaptic studies (rectangular tiles, merged only; scale bar indicates 10 μm). Hoechst stains nuclei; anti-GAD65/67 immunostains inhibitory neurons and synapses; anti-MAP2 immunostains dendrites; and anti-VGLUT1 immunostains excitatory neurons and synapses. **d** The percentage of excitatory neurons in injured networks treated with BDNF significantly decreases compared to control networks (*p* = 0.016) and untreated injured networks (*p* = 0.002). **e** The percentage of inhibitory neurons in injured networks treated with BDNF significantly increases compared to control networks (*p* = 0.016) and untreated injured networks (*p* = 0.002). **f** The E/I synapse balance increases significantly for injured networks (30g 0B and 30g 50B) compared to uninjured networks (0g 0B and 0g 50B; *p* < 0.05 for all comparisons). Treated injured networks are also significantly different than untreated injured networks (*p* = 0.041). 0g = no glutamate; 30g = 30 µM glutamate; 0B = no BDNF; 50B = 50 ng/ml BDNF. For **d** and **e**, *p* values calculated by the Kruskal–Wallis test followed by the Tukey–Kramer multiple comparisons test (*p < 0.05, **p < 0.01), and *n* = 18 datapoints with each representing a field of view. For **f**, *p* values calculated by one-way ANOVA followed by the Tukey–Kramer multiple comparisons test (*p < 0.05), and *n* = 7 datapoints with four fields of view averaged per coverslip. For **d**–**f**, *N* = 3 independent experiments. Gray boxes represent 95% CIs, and solid black lines represent mean. *N* indicates number of experiments, and *n* indicates the number of datapoints.

Glutamate-induced excitotoxicity occurs after a number of injury conditions, including traumatic brain injury, stroke, and neurodegenerative disease^38^. To assess whether treatment with BDNF is neuroprotective, 50 ng/ml BDNF was applied to mature networks (DIV 14) after they were injured with excess glutamate (Fig. 5a). Applying BDNF treatment after injury represents a clinically relevant timeline. We first performed experiments to identify concentrations of glutamate and the length of injury that would result in sublethal injury of hippocampal networks (Supplementary Fig. 2b). Based on our results (Supplementary Fig. 2c), we observed that concentrations of 100 μM glutamate and greater severely decreased network activity. Thus, we chose to injure networks for 30 min with 30 μM glutamate. Furthermore, we chose to use BDNF at 50 ng/ml as the recovery treatment since this BDNF concentration promoted better maintenance of local efficiency and synchrony at 7d post-treatment (Figs. 2c and 3d).

On DIV 14, no injury (vehicle; 0g) or a mild injury (30 μM glutamate; 30g) was applied to mature hippocampal networks for 30 min. Then, networks were either treated with 0B or 50B for 72 hours (Fig. 5a). Prior to analyzing network activity, we quantified whether post-injury treatment with BDNF prevents cell death or loss of dendrites—both known to occur after glutamate-induced excitotoxicity^31,32^—at 0, 24, and 72 hours into the recovery period (“pre”, “24h post”, and “72h post”, respectively). RM ANOVA analysis reveals a significant effect of timepoint (*F* = 54.7 for cell death and *F* = 28.7 for dendrites, both *p* < 0.001) but not of treatment condition or of the interaction of treatment condition and timepoint. Multiple comparisons testing confirms that, while differences exist among timepoints, BDNF treatment does not ameliorate cell death or loss of dendrites promoted by glutamate-induced injury (Supplementary Fig. 4), suggesting that the effects of BDNF on recovery are not due to neuroprotection of these metrics. We dissected these results further and determined how glutamate-induced excitotoxicity and BDNF treatment affect the balance of excitatory-to-inhibitory (E/I) neurons and E/I synapses at 72 hours into the recovery period (schematic and representative images in Fig. 5b, c). We found that treatment with BDNF significantly changes the balance of E/I neurons (Fig. 5d, e), suggesting that BDNF treatment preferentially protects inhibitory neurons. Moreover, we found that glutamate-induced excitotoxicity significantly disturbs the balance of E/I synapses and that BDNF helps partially restore E/I synapse balance back to control levels (Fig. 5f). Our results further indicate that inhibitory synapses are more sensitive to glutamate-induced excitotoxicity (Supplementary Fig. 5d, e) and that the partially restored E/I synapse ratio is instead due to the protection of inhibitory neurons (Fig. 5d, e).

### Treatment with 50 ng/ml BDNF does not prevent injury-induced decreases to network activity

**Fig. 6:**
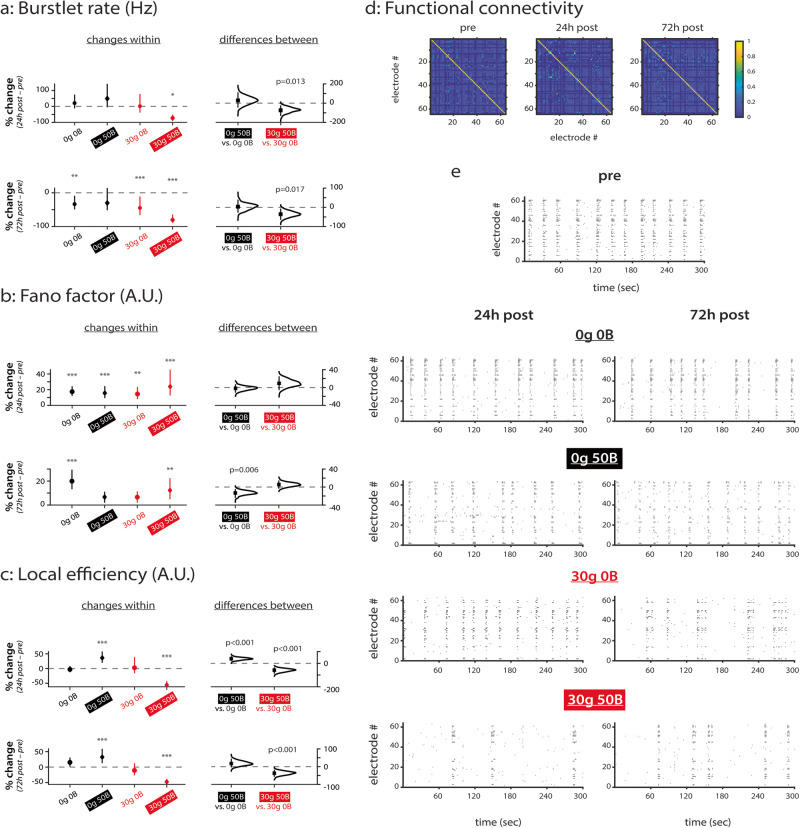
BDNF treatment enhances injury-mediated decreases in network activity. **a** Changes in burstlet rate (Hz). *e* = 92 for 0g 0B; *e* = 85 for 0g 50B; *e* = 91 for 30g 0B; *e* = 64 for 30g 50B. **b** Changes in Fano factor (A.U.). *e* = 266 for 0g 0B; *e* = 315 for 0g 50B; *e* = 265 for 30g 0B; *e* = 193 for 30g 50B. **c** Changes in local efficiency (A.U.). e = 268 for 0g 0B; *e* = 259 for 0g 50B; *e* = 243 for 30g 0B; *e* = 236 for 30g 50B. **d** Functional connectivity matrices used for calculating local efficiency. Shown are representative matrices at each timepoint for the control condition. Colorbar represents strength of cross-correlation. **e** Representative raster plots for each condition. 0g = no glutamate; 30g = 30 µM glutamate; 0B = no BDNF; 50B = 50 ng/ml BDNF. Data in **a**–**d** from *N* = 4 independent experiments. For **a**–**c**: *y*-axis indicates percent change from baseline (pre-injury). Top row compares pre-treatment to 0d post-treatment, and bottom row compares pre-treatment to 7d post-treatment. Plots on left indicate means with 95% CIs, and *p* values calculated by RM ANOVA followed by Tukey–Kramer multiple comparisons test, where *p < 0.05, **p < 0.01, ***p < 0.001 and indicates significant differences between timepoints within the same condition. Distributions on right with means (black squares) and 95% CIs (vertical lines) show comparisons between conditions and were calculated via estimation statistics with p values calculated directly from CIs. *N* indicates number of experiments, and *e* indicates number of electrodes.

Given the effect of BDNF on E/I neuron and E/I synapse ratios (Fig. 5), we next tested whether BDNF can provide neuroprotection at the network level. We recorded spontaneous activity prior to injury at DIV 14 (“pre”), at 24 hours into the recovery period on DIV 15 (“24h post”), and at 72 hours into the recovery period on DIV 17 (“72h post”; Supplementary Fig. 2d). Then we analyzed changes to network dynamics (Fig. 6; all data points shown in Supplementary Fig. 6a–c). We did not record activity immediately after treatment (0 h post) since there was no change in cell death at this timepoint (Supplementary Fig. 4c). We use the same strategies as earlier when analyzing and presenting our MEA data. Moreover, to simplify RM ANOVA analysis, we divided the treatment conditions into one set of four categories (uninjured untreated 0g 0B, uninjured treated 0g 50B, injured untreated 30g 0B, and injured treated 30g 50B) because we cannot discount common signaling pathways between glutamate-induced injury and BDNF recovery^39,40^.

When examining overall network activity via burstlet rate, RM ANOVA reveals that treatment condition (*F* = 4.19, *p* = 0.006), timepoint (*F* = 12.2, *p* < 0.001), and their interaction (*F* = 2.51, *p* = 0.021) all have significant effects (Supplementary Table 2). Through multiple comparisons testing, we find that injury alone has no effect on burstlet rate at 24h post-injury and significantly decreases burstlet rate at 72h post-injury (44%, *p* < 0.001) compared to baseline (30g 0B condition in Fig. 6a, plots on left in both panels, 24h post vs. pre and 72h post vs. pre). Estimation statistics reveal that combining injury with BDNF treatment (30g 50B condition) further decreases activity (over the untreated injured condition 30g 0B) by an additional 75% (*p* = 0.013) at 24h post-injury and by an additional 36% (*p* = 0.017) at 72h post-injury compared to baseline (Fig. 6a, 30g 50B vs. 30g 0B distribution on right in both panels, 24h post vs. pre and 72h post vs. pre). Thus, the combination of glutamate-induced injury and BDNF treatment is worse than injury alone for network burstlet rate at both 24h and 72h post-injury, However, BDNF treatment of uninjured networks has no effect on burstlet rate when compared to untreated control networks.

We next quantified how excitotoxic injury and BDNF treatment affect spiking variability by measuring Fano factor (Fig. 6b). Similar to developing networks, we find from RM ANOVA that only timepoint significantly affects Fano factor (*F* = 36.9, *p* < 0.001; Supplementary Table 2). From multiple comparisons testing, we find that all networks demonstrate significant increases (17% for 0g 0B, *p* < 0.001; 16% for 0g 50B, *p* < 0.001; 14% for 30g 0B, *p* = 0.002; and 24% for 30g 50B, *p* < 0.001) in spiking variability at 24h post-injury compared to baseline (Fig. 6b, plots on left in top panel, 24h post vs. pre). This increase is maintained for control networks (0g 0B; 20%, *p* < 0.001) and for injured treated networks (30g 50B; 12%, *p* = 0.003), but not for the other conditions, at 72h post-injury compared to baseline (Fig. 6b, plots on left in bottom panel, 72h post vs. pre). Underscoring these findings, estimation statistics reveal that, compared to control networks, BDNF treatment of uninjured networks causes Fano factor to shift towards baseline levels at 72h post-injury (Fig. 6b, *p* = 0.006 for 0g 50B vs. 0g 0B distribution on right in bottom panel, 72h post vs. pre).

The different treatment conditions also have distinct effects on local efficiency (Fig. 6c). Indeed, both treatment condition and the interaction of treatment condition and timepoint significantly affect local efficiency (*F* = 25.8 and *F* = 11.3, respectively; *p* < 0.001 for both; Supplementary Table 2). Representative functional connectivity matrices at all timepoints are shown for the control condition (Fig. 6d). Local efficiency does not change over time in control networks (0g 0B; Fig. 6c). In contrast, multiple comparisons testing reveals that local efficiency increases in BDNF treated uninjured networks (0g 50B) by 36% at 24h post-injury (*p* < 0.001) and by 33% at 72h post-injury (*p* < 0.001) compared to baseline (Fig. 6c, left plots in both panels, 24h post vs. pre and 72h post vs. pre). Moreover, estimation statistics reveal that these changes are significantly different compared to control networks at 24h post-injury (Fig. 6c, *p* < 0.001 for 0g 50B vs. 0g 0B distribution on right in top panel, 24h post vs. pre). Surprisingly, local efficiency does not change after injury (30g 0B) but decreases significantly (56% at 24h post and 48% at 72h post; *p* < 0.001 for both) when injured networks receive BDNF treatment (30g 50B; Fig. 6c, left plots in top and bottom panels, 24h post vs. pre and 72h post vs. pre). When comparing 30g 0B and 30g 50B networks using estimation statistics, changes in local efficiency are significantly different when comparing these conditions at 24h post-injury and 72h post-injury to baseline (Fig. 6c, *p* < 0.001 for 0g 50B vs. 0g 0B and for 30g 50B vs. 30g 0B distribution on right in both panels, 24h post vs. pre and 72h post vs. pre). Taken together, our data suggest that BDNF treatment causes a sustained increase in local efficiency in uninjured networks and a sustained decrease in local efficiency in injured networks. We also quantified global efficiency but found no significant changes after injury or with BDNF treatment (data not shown). Overall, treatment with BDNF enhances injury-induced changes to network parameters (Fig. 6e).

### BDNF treatment partially restores network synchronization after excitotoxic injury

**Fig. 7:**
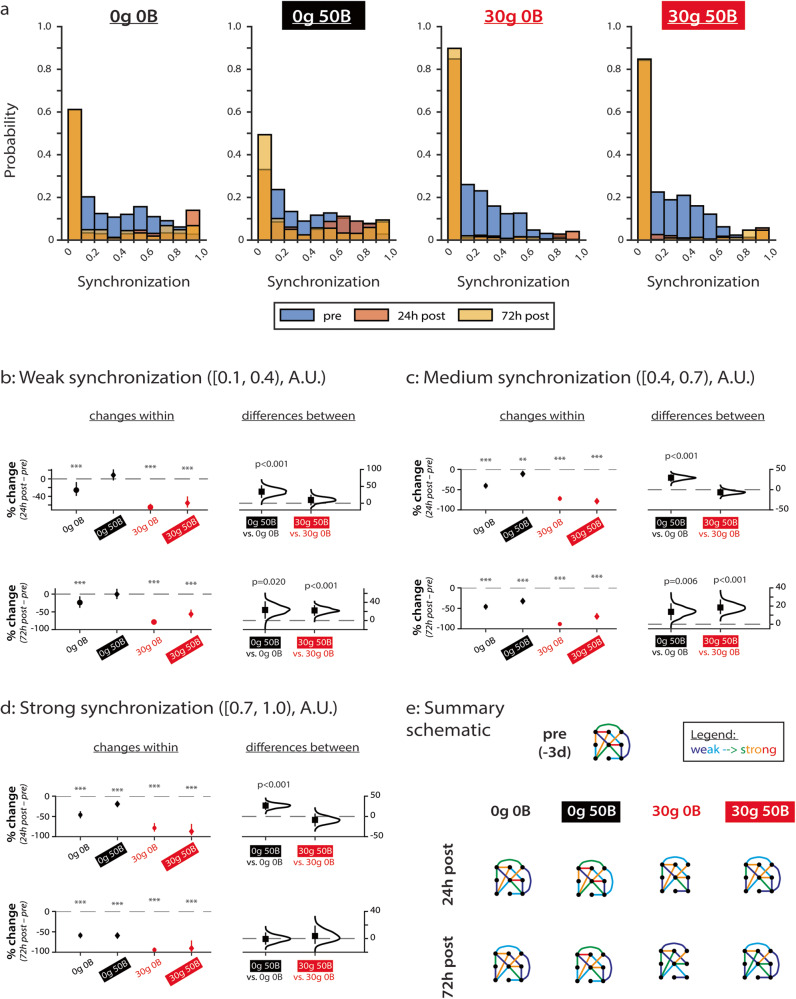
BDNF treatment attenuates glutamate-induced decreases in synchronization of weak and medium connections. **a** Distribution of connection strengths for all networks over time. From left to right: control (0g 0B), 0g 50B, 30g 0B, and 30g 50B. **b** Changes in connections with initially weak synchronization (values of [0.1,0.4)). *e* = 412 for 0g 0B; *e* = 528 for 0g 50B; *e* = 970 for 30g 0B; *e* = 482 for 30g 50B. **c** Changes in connections with initially medium synchronization (values of [0.4,0.7)). *e* = 366 for 0g 0B; *e* = 398 for 0g 50B; *e* = 442 for 30g 0B; *e* = 266 for 30g 50B. **d** Changes in connections with initially strong synchronization (values of [0.7,1.0)). *e* = 170 for 0g 0B; *e* = 224 for 0g 50B; *e* = 80 for 30g 0B; *e* = 26 for 30g 50B. **e** Summary schematic of how synchronization changes during development and with BDNF treatment. Colors correspond to synchronization values, ranging from weak (dark blue) to strong (red). Data in **a**–**d** from *N* = 4 independent experiments. 0g = no glutamate; 30g = 30 µM glutamate; 0B = no BDNF; 50B = 50 ng/ml BDNF. For **b**–**d**: *y*-axis indicates percent change from baseline (pre-injury). Top row compares pre-treatment to 0d post-treatment, and the bottom row compares pre-treatment to 7d post-treatment. Plots on left indicate means with 95% CIs, and *p* values calculated by RM ANOVA followed by Tukey–Kramer multiple comparisons test, where ***p < 0.001 and indicates significant differences between timepoints within the same condition. Distributions on the right with means (black squares) and 95% CIs (vertical lines) show comparisons between conditions and were calculated via estimation statistics with *p* values calculated directly from CIs. *N* indicates the number of experiments, and *e* indicates number of electrodes.

To further understand how BDNF treatment affects network recovery from glutamate-induced excitotoxicity, we again analyzed how synchronization between electrodes changes during the recovery period (Fig. 7; all data points shown in Supplementary Fig. 6d–f). First, we analyzed the distribution of synchronizations for all conditions, and as we observed in younger networks (Fig. 3a), we found a skew towards weak connections in these more mature networks (Fig. 7a). We again use the same analyses for our MEA data as in previous sections.

RM ANOVA reveals that, for all categories of synchronization (weak, medium, and strong), treatment condition (*F* = 58.9, *F* = 102, *F* = 33.6, respectively; all *p* < 0.001), timepoint (*F* = 126, *F* = 822, *F* = 416, respectively; all *p* < 0.001), and the interaction between treatment condition and timepoint (*F* = 31.6, *F* = 62.9, *F* = 26.6, respectively; all *p* < 0.001) significantly affect each category of synchronization (Supplementary Table 2).

Multiple comparisons testing reveals that control networks (0g 0B) show decreased synchronization at 24 and 72 hours after injury compared to pre-injury regardless of initial synchronization strength (>20%, >40%, and >40%, respectively, for weak, medium, and strong connections; *p* < 0.001 for all; Fig. 7b–d, left plots in top and bottom panels; 24h post vs. pre and 72h post vs. pre). Comparatively, using estimation statistics, BDNF treatment of uninjured networks (0g 50B) (i) prevents decreases in synchronization of initially weak connections for 72 hours (Fig. 7b, 0g 50B vs. 0g 0B distribution on right in the top and bottom panels; *p* < 0.001 for 24h post vs. pre and *p* = 0.020 for 72h post vs. pre), (ii) attenuates (by >10%) decreases in synchronization of initially medium connections for 72 hours (Fig. 7c, 0g 50B vs. 0g 0B distribution on right in top and bottom panels; *p* < 0.001 for 24h post vs. pre and *p* = 0.006 for 72h post vs. pre), and (iii) attenuates decreases in synchronization (by 26%) of initially strong connections for 24 hours (Fig. 7d, 0g 50B vs. 0g 0B distribution on right in top panel, *p* < 0.001 for 24h post vs. pre).

BDNF treatment after injury provides partial rescue of certain connection strengths. Estimation statistics reveal that compared to untreated injured networks (30g 0B), BDNF treatment of injured networks (30g 50B) attenuates decreases in weak (by 22%, *p* < 0.001) and medium (by 18%, *p* < 0.001) connections at 72h post-injury compared to pre-injury (Fig. 7b, c, 30g 50B vs. 30g 0B distribution in bottom panels, 72h post vs. pre). For initially strong connections, BDNF treatment does not prevent decreases at either 24 or 72h post-injury compared to pre-injury (Fig. 7d, 30g 50B vs. 30g 0B distribution in top and bottom panels, 24h post vs. pre and 72h post vs. pre).

Taken together, our results suggest that synchronizations of all categories are affected by treatment, timepoint, and their interactions. Importantly, estimation statistics reveal that BDNF treatment attenuates decreases of weak and medium connections for both uninjured (at 24 and 72 hours) and injured (at 72 hours) networks compared to baseline (Fig. 7e).

### Functional network-level analyses suggest that BDNF exerts mixed effects on injured networks

**Fig. 8:**
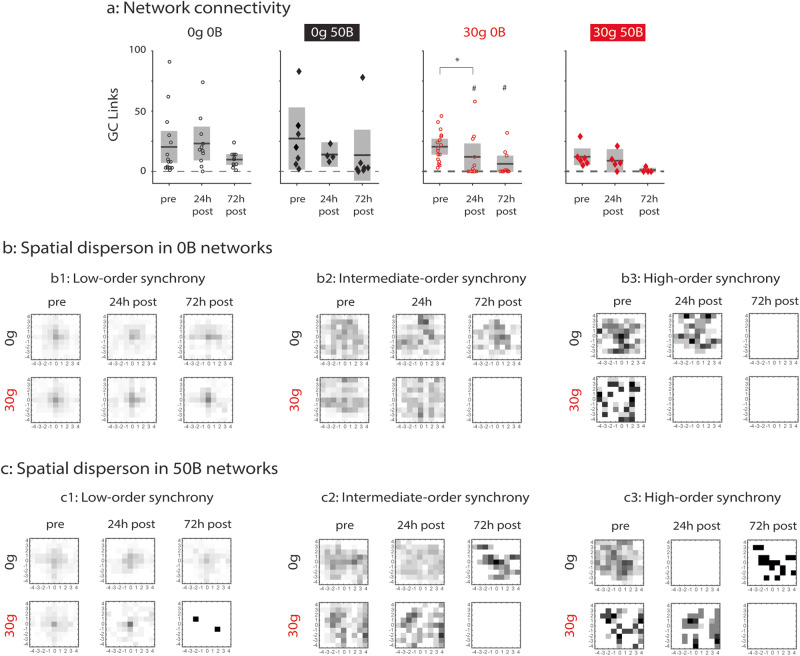
Granger causal and synchrony analyses after glutamate injury and BDNF recovery treatment. **a** From left to right: all GC links in control (0g 0B), 0g 50B, 30g 0B, and 30g 50B networks. *, # *p* < 0.05. Asterisk symbols (*) indicate comparisons between timepoints of the same condition. Hash symbols (#) indicate comparison to 0g 0B condition at same timepoint. Comparisons were made using a two-sided Wilcoxon’s rank sum test. Solid black lines indicate the mean, and gray boxes indicate 95% CIs. **b** Spatial dispersion of synchronous units in 0B networks grouped by low-order (**b**1), intermediate-order (**b**2), and high-order synchrony (**b**3). **c** Spatial dispersion of synchronous units in 50B networks grouped by low-order (**c**1), intermediate-order (**c**2), and high-order synchrony (**c**3). For **b** and **c**, top row is control (0B) networks, and bottom row is injured (30g) networks. Statistically significant *p* values for data in **b** and **c** are included in Supplementary Material and were determined by two-sample KS tests. 0g = no glutamate; 30g = 30 µM glutamate; 0B = no BDNF; 50B = 50 ng/ml BDNF. Data from *N* = 11 independent experiments. 0g 0B: *n* = 19 for pre; *n* = 14 for 24h post; *n* = 13 for 72h post. 0g 50B: *n* = 13 for pre; *n* = 8 for 24h post; *n* = 12 for 72h post. 30g 0B: *n* = 20 for pre; *n* = 14 for 24h post; *n* = 12 for 72h post. 30g 50B: *n* = 11 for pre; *n* = 9 for 24h post; *n* = 8 for 72h post. *N* indicates number of experiments, and *n* indicates number of datapoints (MEA networks).

We next expanded our analyses of the functional network-level effects of BDNF on injured networks by using GC and higher-order synchrony analyses. We first estimated GC networks and counted the number of links as a measure of connectivity (Fig. 8a). We found no difference in connectivity across injury and treatment conditions at baseline. The number of GC links in uninjured networks (0g 0B and 0g 50B) also does not vary significantly across the recording period, indicating that BDNF treatment does not affect homeostasis of network connectivity in uninjured networks. Untreated injured networks (30g 0B) have significantly fewer GC links at 24 hours after injury than at pre-injury (*p* < 0.05, * symbol), whereas this is not the case for BDNF-treated injured networks (30g 50B). As this implies a possible dependency of the effect of BDNF treatment on injury level, we used RM ANOVA to test for such an interaction. Although the effect of time was confirmed (*p* = 0.006), no significant interaction between injury and treatment was found (*p* = 0.215). However, the number of injured in vitro networks that were sufficiently active to perform GC analysis (13 in total, with 7 for 0B and 6 for 50B) was small compared to the number of active uninjured cultures (32 in total, with 15 for 0B and 17 for 50B cultures). Our results thus indicate that, while excitotoxic injury disrupts homeostasis of network connectivity, BDNF treatment may protect connectivity in injured networks within 24 hours.

We next sought to characterize how BDNF treatment affects higher-order synchrony in recovering injured networks. To this end, we investigated how the spatial extent of synchronized electrodes changed by condition and across time; these distributions were obtained by compiling over groups of synchronous electrodes. We first considered untreated networks that were uninjured (0g 0B) or injured (30g 0B), comparing spatial distributions of low-order (Fig. 8b1), intermediate-order (Fig. 8b2), and high-order (Fig. 8b3) synchronous electrodes using two-sample KS tests.

For low-order synchronous electrodes (Fig. 8b1), spatial distributions do not change significantly in either control or injured networks over time (24h post vs. pre: *p* = 0.672 for 0g 0B and *p* = 0.848 for 30g 0B; 72h post vs. 24h post: *p* = 0.672 for 0g 0B and *p* = 0.848 for 30g 0B). For control networks (0g 0B; top row), the spatial distributions of intermediate-order synchronous electrodes (Fig. 8b2) and high-order synchronous electrodes (Fig. 8b3) become significantly more concentrated between pre-injury and 24 hours after injury (*p* = 0.029 and *p* < 0.001, respectively). Between 24 and 72 hours after injury, intermediate-order synchronous electrodes become more dispersed (*p* = 0.005) whereas high-order synchronous electrodes are not detected at 72 hours after injury. For untreated injured networks (30g 0B, bottom row), similar to control networks, the spatial distribution of intermediate-order synchronous electrodes becomes more concentrated at 24 hours after injury (*p* < 0.001), but in contrast to control networks, no intermediate-order synchronous electrodes are detected at 72 hours after injury. Moreover, no high-order synchronous electrodes are detected at 24 hours or at 72 hours after injury for damaged networks. These results suggest that groups of synchronous electrodes are more susceptible to excitotoxic injury as the size of the group increases. Hence, we hypothesize that, if BDNF treatment were neuroprotective for this metric, then the spatial distributions of intermediate- and high-order synchronous electrodes would be more stable over time.

We next compared the spatial distributions of synchronous electrodes in uninjured and injured networks that received BDNF treatment (Fig. 8c). BDNF treatment does not change the spread of low-order synchronous electrodes (Fig. 8c1) in uninjured networks (0g 50B; top row) between pre-injury and 24 hours after injury (*p* = 0.198) or between 24 hours and 72 hours after injury (*p* = 0.133). For intermediate-order synchronous electrodes (Fig. 8c2), there is a significant increase in the spread between pre-injury and 24 hours after injury (*p* = 0.008) followed by a significant decrease between 24 and 72 hours after injury (*p* = 0.032), contrasting the trend for control networks (0g 0B; Fig. 8b2, top row). However, for high-order synchrony (Fig. 8c3), uninjured networks that received BDNF treatment (0g 50B; top row) have no groups of high-order synchronous units at 24 hours after injury and only one detected group at 72 hours after injury, suggesting faster degradation of high-order synchrony than in control networks (0g 0B; Fig. 8b3, top row).

BDNF treatment exerts mixed effects on synchronous electrodes after excitotoxic injury. The spread of low-order synchronous electrodes (Fig. 8c1) in injured networks (30g 50B; bottom row) does not differ from pre-injury to 24 hours after injury (*p* = 0.812), but only one group of low-order synchronous electrodes is detected at 72 hours after injury, suggesting that BDNF treatment is actually harmful for low-order synchrony in injured networks (30g 50B). In contrast, for intermediate-order synchronous electrodes (Fig. 8c2), there is no change in spread between pre-injury and 24 hours after injury (*p* = 0.609), and at 72 hours after injury, there are no detected groups of intermediate-order synchronous electrodes, mirroring the trends in untreated injured networks (30g 0B; Fig. 8b2, top row) and indicating that BDNF treatment had no effect on intermediate-order synchronous electrodes. High-order synchrony (Fig. 8c3), in contrast, is protected in the short term in BDNF-treated injured networks (30g 50B; bottom row) because, compared to untreated injured networks (30g 0B), high-order synchronous units were detected at 24 hours after injury with similar spread as at baseline (*p* = 0.171).

Together, GC and higher-order synchrony analyses show that BDNF treatment exerts mixed effects on functional network-level properties in recovering injured networks. First, GC analyses reveal that BDNF treatment protects injured networks within 24 hours of injury. Moreover, in injured networks, BDNF treatment disrupts low-order synchrony at 72 hours after injury, has no effect on intermediate-order synchrony, and protects high-order synchrony at 24 hours after injury. These results suggest that BDNF treatment partially promotes network homeostasis after injury by exerting complex actions that somewhat mitigate the effects of excitotoxic injury.

### In silico simulation of glutamate injury and BDNF treatment suggests that BDNF indirectly influences excitatory synaptic strength

**Fig. 9:**
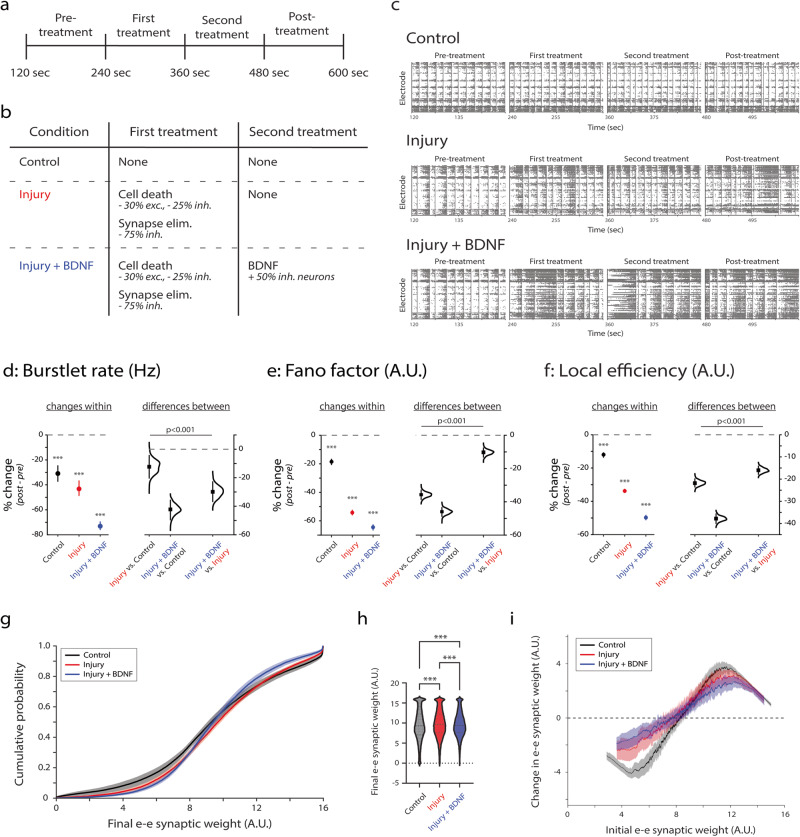
In silico networks that have similar activity compared to in vitro networks reveal BDNF-mediated remodeling of excitatory synapses. **a** Simulation timeline showing the order and duration of treatment epochs. **b** Description of treatments at each interval for each condition. **c** Representative raster plots for each condition showing the first 30 s of each interval. **d** Changes in burstlet rate (Hz). *e* = 292 for Control; *e* = 286 for Injury; *e* = 275 for Injury + BDNF. **e** Changes in Fano factor (A.U.). *e* = 354 for Control, Injury, and Injury + BDNF. **f** Changes in local efficiency (A.U.). *e* = 354 for Control; *e* = 341 for Injury; *e* = 340 for Injury + BDNF. For **d**–**f**: *y*-axis indicates percent change from baseline (pre-treatment). Plots on left indicate means with 95% CIs, and *p* values calculated by RM ANOVA followed by Tukey–Kramer multiple comparisons test, where ***p < 0.001 and indicates significant differences between timepoints within the same condition. Distributions on the right with means (black squares) and 95% CIs (vertical lines) show comparisons between conditions and were calculated via estimation statistics with *p* values calculated directly from CIs. **g** Mean ± SEM for cumulative distribution functions for each condition, where ***p < 0.001 for comparison of all conditions via two-sample Kolmogorov–Smirnov test. **h** Average excitatory-excitatory synaptic weight during the post-treatment epoch across all conditions, where ***p < 0.001 calculated via one-way ANOVA followed by Tukey–Kramer multiple comparisons test. **i** Moving average ± SEM (window size = 250) for average excitatory-excitatory synaptic weight during the post-treatment epoch minus average excitatory–excitatory synaptic weight during pre-treatment epoch as a function of average excitatory–excitatory synaptic weight during pre-treatment epoch. For **g**–**i**: Total number of synapses *s* = 50523 for Control, *s* = 25011 for Injury, and *s* = 24762 for Injury + BDNF. *N* = 6 independent simulations. For all data in **d**–**i**, *s* indicates number of synapses, and *e* indicates number of electrodes.

To investigate the mechanism by which BDNF returns injured networks to homeostasis, we developed an in silico neuronal network model (Fig. 9a) based on the work of Masquelier and Deco^41^. In particular, we modified and extended their model and our previous work^17^ to better match the specific characteristics of our in vitro neuronal cultures, including neuronal E/I balance (Fig. 5d, e), synaptic E/I balance (Fig. 5f), and neuronal density (see “Methods” section: subsection “Primary neuronal dissections and cell culture”). We modeled glutamate injury as cell death in the excitatory and inhibitory compartments at a rate of 30% and 25%, respectively, along with all their synapses, and we further reduced the number of inhibitory synapses by 75% (Supplementary Fig. 7a–c). This yielded a net reduction in inhibitory synapses of 86% (Supplementary Fig. 8c, d), consistent with our in vitro cultures (Fig. 5f and Supplementary Fig. 5). We notably scaled back the level of cell death relative to in vitro findings to compensate for cell loss associated with low plating density (see the “Methods” section: subsection “Primary neuronal dissections and cell culture”). We modeled BDNF as a 50% recovery of inhibitory neurons, along with their original connections, consistent with our in vitro findings. Because we modeled BDNF as a recovery of previously injured neurons, we did not simulate BDNF treatment in the absence of injury (Fig. 9b, c), resulting in the simulated conditions Control, Injury, and Injury + BDNF to match the in vitro conditions 0g 0B, 30g 0B, and 30g 50B, respectively.

By faithfully representing glutamate- and BDNF-mediated structural changes to excitatory and inhibitory neurons and synapses, we reproduced several of the functional activity properties displayed by the in vitro networks (Fig. 9; all data points shown in Supplementary Fig. 9). For all parameters (burstlet rate, Fano factor, local efficiency), RM ANOVA reveals that treatment condition, timepoint, and their interaction significantly affects the post-treatment behavior of the networks compared to pre-treatment (refer to Supplementary Table 3 for *F*-statistics and associated p values). Multiple comparisons additionally reveal a significant decrease in burstlet rate for injury alone (“Injury”) and injury with BDNF treatment (“Injury + BDNF”), consistent with in vitro findings (Fig. 9d vs. Fig. 6a). While we observe a decrease in Fano Factor in injured networks compared to control, the decrease we observe for Injury + BDNF networks compared to injury alone is inconsistent with in vitro findings (Fig. 9e vs. Fig. 6b). We attribute these differences to the lack of asynchronous firing in our in silico networks (Fig. 9c) compared to in vitro networks (Fig. 6e). When comparing local efficiency, in silico we match the trend of decreasing efficiency with Injury networks compared to Injury + BDNF networks (Fig. 9f vs. Fig. 6c).

We next extended our computational model to understand how BDNF affects synaptic-level remodeling in the network. To that end, we examined spike-timing-dependent plasticity (STDP)-mediated changes in excitatory-to-excitatory (e–e) synaptic strength following injury with and without BDNF treatment. In general, we found that both Injury and Injury + BDNF modify the cumulative probability distribution of excitatory synaptic weight (Fig. 9g, *p* < 0.001 via two-sample Kolmogorov–Smirnov test), narrowing the dispersion of synaptic weights and limiting the number of synapses at the extremes. We additionally find that injury increases the average excitatory synaptic weight post-injury compared to control (Fig. 9h, mean value of 9.84 vs. 9.49; *p* < 0.001 via one-way ANOVA followed by multiple comparisons testing), as does injury with BDNF treatment (Fig. 9h, mean value of 9.64 vs. 9.49; *p* < 0.001 via one-way ANOVA followed by multiple comparisons testing). As a result, BDNF treatment following injury partially restores the average excitatory synaptic weight in the network back to control levels relative to injury (Fig. 9h, mean value of 9.64 vs. 9.84; *p* < 0.001 via one-way ANOVA followed by multiple comparisons testing). To understand which synapses are affected by injury and BDNF, we examined the change in excitatory synaptic weight as a function of original excitatory synaptic weight (Fig. 9i). In the control condition, weak excitatory synapses become weaker and strong excitatory synapses become stronger over time, rather than weak synapses becoming stronger, to drive the increase in overall excitatory synaptic weight. We find that both injury alone and Injury + BDNF not only limit the ability of weak synapses to become weaker but also prevent strong synapses from becoming stronger, with BDNF treatment after injury more greatly limiting changes to excitatory synapse weight. Following injury (with or without BDNF), even weak synapses must be recruited to maintain “normal” bursting activity. In particular, given that BDNF treatment directly affects inhibitory neurons, it is striking that it has this effect on excitatory-to-excitatory synapses following injury. This finding suggests that, by protecting inhibitory neurons, BDNF also protects important inhibitory-to-excitatory synapses that indirectly allow BDNF to influence the excitatory-to-excitatory synapses that drive network activity.

Taken together, these results suggest that when we directly simulate the structural effects of injury and BDNF on excitatory and inhibitory neurons and synapses, we can reproduce the key finding that BDNF has a limited therapeutic effect on network-based measured activity in injured networks. In turn, we further find that this limited therapeutic effect may be related to the indirect effect of BDNF on excitatory remodeling to prevent extreme synaptic weights and maintain weak excitatory synapses.

## Discussion

In this work, we employ MEA analysis, imaging, and simulations to perform a detailed study of how developing and injured networks respond to treatment with BDNF. In general, we find that the additive effects of BDNF on injury are mixed: BDNF clearly worsens some effects of injury but promotes homeostasis for other aspects of network dynamics. On the one hand, BDNF treatment preserves synchronization of weak and medium connections at 72h post-injury, network connectivity at 24h post-injury, and inhibitory neurons at 72h post-injury. On the other hand, BDNF treatment enhances injury-induced decreases in burstlet rate and disrupts low-order synchrony after excitotoxic injury. It is, of course, possible that these short-term changes, regardless of whether we interpret them to be “helpful” or “harmful”, do indeed promote longer-term network homeostasis and functional recovery (e.g., weeks or months after injury).

Are the mixed effects of BDNF treatment on the functional recovery of hippocampal networks due to inherent plasticity in our networks? Although in vitro neuronal networks are considered to be mature by DIV 14—the time at which we induced chemical injury—the networks are still developing. BDNF is known to be a positive regulator of both dendritic and synaptic plasticity^12,26,28,42^, and thus, it is possible that inherently plastic networks that are both injured with excess glutamate and treated with BDNF may result in “hyperplasticity,” causing an abnormal network. Indeed, it has been suggested that excessive plasticity could be disadvantageous for functional recovery^43^. However, our imaging and modeling results suggest a more precise effect of BDNF, preferentially preserving inhibitory neurons and affecting excitatory synaptic plasticity through its protection of inhibitory-to-excitatory synapses. Rather than a uniform effect of BDNF across the circuit in vitro, our results demonstrate a more nuanced remodeling process during development and after injury. Moreover, since it was necessary to perform our imaging studies at a much lower density than our MEA studies, it is possible that the effects of glutamate-induced excitotoxicity on cell death and on the excitatory-to-inhibitory neuronal and synaptic balances are exaggerated.

What do our findings mean for BDNF as a treatment for injuries or neurological disorders that are characterized by glutamate-induced excitotoxicity? BDNF signaling has been proposed as a mechanism for promoting synaptic repair in neurodegenerative diseases (reviewed in ref. ^44^) because BDNF levels are often lower in pathophysiological conditions^45^. Despite promising initial studies, such as the successful restoration of BDNF protein levels in mouse models of Alzheimer’s Disease^46^ and Parkinson’s Disease (reviewed in ref. ^47^), BDNF treatment has not yet been successful clinically for patients suffering from these or other neurodegenerative diseases^48^. Moreover, for spinal cord injury, a combination of BDNF treatment with olfactory ensheathing cell transplantation resulted in impaired motor recovery^49^, indicating that the actions of BDNF are more complex than only promoting synaptic plasticity. The differences in TrkB receptor phosphorylation at 0d post- and 7d post-treatment (Fig. 1b, c) may also explain some of the differences in BDNF-promoted changes to network dynamics. The lower concentration is thought to target TrkB specifically, whereas the higher concentration likely also activates TrkA and TrkC^27,29,33^. Future work will investigate in more depth how time-dependent TrkB activation affects hippocampal network function and may be involved in recovery. Indeed, one study has indicated that network recovery begins quickly, as soon as 15 min, after injury^50^.

To better elucidate the complex actions of BDNF during development and after injury, we adapted a recently developed likelihood model-based approach to infer higher-order network synchrony^35,36^. Unlike model-free cross-correlational analyses that are restricted to pairwise comparisons^51^, likelihood models can characterize synchrony amongst more than two neurons^52^. Previous approaches, however, lack an exact inference procedure to identify the significance of higher-order synchrony in single-trial data. Building on marked point process models^52^ for ensemble spiking activity, the approach in our recent work^35,36^ establishes an exact statistical inference framework that is used to identify significantly correlated activity between an arbitrary number of units. Characterizing network synchrony in addition to GC analysis allows us to quantify network-level properties of hippocampal neuronal networks more thoroughly as they develop and recover from injury in vitro. Indeed, the combined GC and synchrony analyses, which are novel for MEA data, allowed us to reveal that the 25B and 50B treatments have opposite effects on network connectivity and higher-order synchrony.

In this work, we also developed an in silico neuronal network model to reproduce the in vitro findings and develop a more nuanced view of how BDNF affects network homeostasis post-injury. One limitation of our in silico model was the more prominent bursting behavior in this model compared to in vitro networks (Fig. 9c). Our groups previously showed that, despite this “burstiness,” the network model can be tailored to successfully match activity metrics, such as burstlet rate and local efficiency, but less faithfully reproduces metrics that measure subtle variation in firing across electrodes, such as Fano factor^17^. Despite this limitation, our model captures the important overall effects of BDNF and glutamate application.

Our in silico model suggests that, in this system, BDNF indirectly affects excitatory synapse remodeling and, in doing so, can only partly restore network-level outcomes after injury. Given that, in our in silico model, we modeled BDNF as only directly affecting inhibitory neuron survival, consistent with our in vitro data, its effect on excitatory-to-excitatory synaptic plasticity was unexpected. It is possible that the effects BDNF exerts on synchronization after injury—attenuating decreases in weak and medium connections at 72h post injury—are related to these findings: BDNF preserves weak and medium connections between electrodes (groups of neurons), and the synaptic level effect is to limit changes to excitatory synaptic weight after injury, thus preserving network homeostasis. Through our modeling process, we also noticed that if inhibitory synapse loss by injury was too great, BDNF was unable to affect both the activity and excitatory synapse plasticity, underscoring the importance of minimum inhibitory tone in the network to support action by BDNF (data not shown).

There is debate in the literature regarding which types of connections BDNF affects and under which conditions. Although studies demonstrate that BDNF application enhances inhibitory neurotransmission^13,26,53^, others show that BDNF application decreases the efficacy of inhibitory neurotransmission^54,55^. Moreover, multiple groups have reported BDNF-mediated increases in excitatory neurotransmission^26,53,56^. Some groups have sought to reconcile these differences, examining the differences in BDNF overexpression compared to bath application^56^, in treatment with pro-BDNF compared to mature BDNF^57^, or in dose-dependence^58,59^ as we included in our study, but there is still a large degree of uncertainty regarding the diverse effects mediated by BDNF. Interestingly, glutamatergic synapse activity has been shown to induce BDNF-dependent potentiation of γ-aminobutyric acid (GABA)-ergic synapses^60^, supporting our hypothesis that, in this system, BDNF increases inhibitory connections. By virtue of our interdisciplinary approach, we are able to weigh in regarding the current controversy of what types of synapses BDNF acts upon, and we present data that shed new light on how BDNF might directly affect inhibitory synapses and indirectly affect excitatory synapses.

In addition to our hypothesis that BDNF primarily affects inhibitory neurons and connections, there are several other possible explanations we did not pursue. For example, as primary neuronal networks develop in vitro, inhibition will emerge, and neurons will undergo a “GABA switch” by DIV 7^61^. In the dose–response portion of this study (Figs. 1–4), it is possible that our treatment of networks with BDNF from DIV 7–10 coincided with this emergence of inhibition and presented some confounding of our results. In particular, the “GABA switch” could be responsible for the decreases in synchronization observed between the pre-treatment timepoint (DIV 7) and the first post-treatment timepoint (DIV 10). Because our goal was to align the timing of this study with our previous work^27^, we did not attempt to treat networks with BDNF definitively before or after the “GABA switch”, as other studies have done^62^.

We were also intrigued by our finding that, over time, local efficiency is increased compared to baseline levels in control networks (Fig. 2c). A higher local efficiency means that local neighbors can process information more effectively, which would be advantageous as a network matures in vitro, and is characteristic of the network becoming more small-world^63–65^. Our results potentially indicate that increased local efficiency over time is an intrinsic feature of in vitro neural networks that is disturbed by the higher concentration of BDNF (immediately after treatment ends) and by the lower concentration of BDNF (at 7 days after treatment ends). Conversely, global efficiency was not an effective metric for our networks, likely due to the poor spatial resolution of MEAs, but other work has shown that it could be a warning for the decline of network function since an increase in global efficiency has been shown to follow network injury^66^.

In summary, we show that network-level homeostasis can be described by an array of features, ranging from characteristics of electrode activity to the interactions across electrodes, each of which is susceptible to disruption by glutamate-induced excitotoxicity. Due to its prevalent roles in neural growth and synaptic regulation, we investigated how BDNF treatment promotes homeostasis in developing hippocampal networks by analyzing the effects of treatment on network features. We found that BDNF treatment limited widespread communication across networks and exerted mixed effects as a post-injury treatment. BDNF-mediated effects were often insufficient to return injured networks to their pre-injury states, as our GC and higher-order synchrony analyses and simulations suggest that the network-level effect of BDNF is to promote inhibitory connections. It is possible that the subtle preservation of homeostasis elicited by BDNF to high-order synchrony or the synchronization of weak and medium connections—but not to burstlet rate or local efficiency or low-order synchrony—are critical factors for the long-term functional recovery of networks.

Here we used monolayer culture of primary rat embryonic hippocampal neurons. Other studies have examined how physical constraints affect functional organization^67,68^ or how aggregates of neurons interact and display resilience after specific nodes are removed^69^. Future work by our laboratory to investigate network injury and recovery will take inspiration from these and other recent studies^70^. Finally, we also intend to investigate longer-term outcomes of BDNF treatment after injury. Importantly, this study provides new insights into the complex molecular mechanisms and interactions that underlie the functional properties characterizing network homeostasis and suggests that shifting homeostatic set points would require equally complex and subtle interventions.

## Methods

### Primary neuronal dissections and cell culture

Neuronal cultures were prepared from the hippocampi of Sprague Dawley rat embryos at 18 days of gestation (E18) as described previously^71^. The hippocampi were dissociated using manual trituration, and we did not distinguish between hippocampi from male and female embryos. For Western blot analysis and imaging experiments, cells were plated onto PDL-coated plastic 35 mm dishes or onto PDL-coated 12 mm glass coverslips within 24-well plates, respectively, at a density of ~850 cells/mm^2^. For microelectrode array (MEA) experiments, cells were plated onto PDL- and laminin-coated MEAs at a density of 1 × 10^6^ cells per MEA (3.5 × 10^3^ cells/mm^2^) as we previously reported^15–18^. It was necessary to plate cells at a much lower density for Western blot experiments because of the number of conditions and timepoints per experiment for imaging studies to ensure the analysis was feasible. Cultures were kept in a humidified 37 °C incubator with 5% CO_2_ and maintained in NbActiv4 medium (Brain Bits, cat. no. Nb4-500), which contains Neurobasal medium, B27, glutamine, creatine, estrogen, and cholesterol^72^. Additionally, 1% penicillin–streptomycin (Thermo Fisher, cat. no. 15140122) was added to the culture medium to prevent contamination. Half of the culture medium was changed every other day.

All studies involving animals were performed in accordance with and received ethical approval by the Institutional Animal Care and Use Committee (IACUC) at Rutgers University. We have complied with all relevant ethical regulations for animal use.

### Western blot analysis

Hippocampal neurons cultured in 35 mm dishes were treated with BDNF according to the schedule in Supplementary Fig. 2a. At the appropriate timepoint, cultures were scrape harvested into RIPA buffer [50 mM Tris–HCl pH 7.4; 150 mM NaCl; 0.5% deoxycholate; 1% NP-40; 1 mM EDTA pH 7.4; 0.1% sodium dodecyl sulfate (SDS)] containing 1 mM phenylmethylsulfonyl fluoride, 1× PhosSTOP phosphatase inhibitor tablet (MilliporeSigma, cat. no. 4906837001), and 1× Protease Inhibitor Cocktail (MilliporeSigma, cat. no. 11697498001). Protein concentrations were measured with the Pierce BCA Protein Assay Kit (ThermoFisher, cat. no. 23225), and 20 μg of protein extracts were resolved on 10% acrylamide gels by SDS–PAGE, transferred to polyvinylidene difluoride membranes, and blocked with 5% bovine serum albumin (BSA) in TBST (20 mM Tris pH 7.5; 150 mM NaCl; 0.1% Tween-20) for 1 h. Membranes were incubated with rabbit anti-TrkB antibody (1:1000; Cell Signalling Technology, cat. no. 4603T, clone 80E3), which detected both TrkB (~90 kDa) and phospho-TrkB (~140 kDa), in 5% BSA in TBST overnight at 4 °C. Membranes were washed with TBST and incubated with goat anti-rabbit HRP-conjugated secondary antibody (1:250; Rockland Immunochemicals, Inc., cat. no. 611-1302). Chemiluminescence signals were detected on the LI-COR Odyssey Fc Imaging system (LI-COR Biosciences) with Immobilon Western Chemiluminescent HRP Substrate (MilliporeSigma, cat. no. WBKLS0100). Phospho-TrkB quantification was compared to total TrkB. Six separate trials were performed.

### Cell death experiments: immunofluorescence, imaging, and analysis

Hippocampal neurons cultured on 12 mm glass coverslips were subjected to glutamate injury and BDNF treatments shown in Supplementary Fig. 2d and fixed in 4% paraformaldehyde in phosphate-buffered saline at the timepoints shown. An additional timepoint immediately after injury (0 h) was added to capture the initial effects of glutamate-induced excitotoxicity that we were unable to observe with MEA recordings. After fixation, the immunostaining protocol in our previous work was followed^27,29,30^. The primary antibody was mouse anti-MAP2 (1:1000; BD Biosciences, cat. no. 556320, clone Ap20), and the secondary antibody was donkey anti-mouse AlexaFluor 488 (1:250; Thermo Fisher, cat. no. A32766). Hoechst 33342 (at 2 μg/ml; Millipore Sigma, cat. no. B2261) was used to mark nuclei, and coverslips were mounted on glass microscope slides using Fluoromount G (Fisher Scientific, cat. no. OB100-01).

Coverslips were imaged on an EVOS FL microscope (Thermo Fisher) using a ×10 objective. Four images were taken per coverslip of wavelengths at 488 and 405 nm, and each condition had three coverslips. Three separate trials were performed. For cell death quantification, the number of MAP2-positive cells was counted per field of view and averaged for each condition and for each trial. For dendrite quantification, the MAP2 images were filtered using a rotating, two-dimensional Laplacian of Gaussian filter to preferentially extract the long, thin structure of dendrites^73,74^. The filtered MAP2 image was thresholded to produce a binary image. The nuclei images were also thresholded to produce a binary image. To remove nuclei from the dendrite images, the thresholded nuclei image was subtracted from the thresholded MAP2 image, leaving only the dendrites. The final image was skeletonized using the MATLAB function bwskel. The number of detected pixels represents the total dendrite length and was compared across each of the conditions.

### Excitatory/inhibitory neuron and synapse ratio experiments: immunofluorescence, imaging, and analysis

As with our cell death experiments, hippocampal neurons cultured on 12 mm glass coverslips were subjected to glutamate-induced injury and BDNF treatments shown in Supplementary Fig. 2d. Coverslips were fixed in 4% paraformaldehyde in phosphate-buffered saline at 72h post-injury timepoint (DIV 17). We again followed the immunostaining protocol in our previous work^27,29,30^. The primary antibodies used were the following: polyclonal chicken anti-MAP2 (1:1000; Novus Biologicals, cat. no. NB300-213), mouse anti-VGLUT1 (1:250; Synaptic Systems, cat. no. 135 011, clone 68B7), rabbit anti-GAD65/67 (1:250; Abcam, cat. no. ab183999, clone EPR19366). The secondary antibodies used were the following: goat anti-rabbit IgG AlexaFluor 488 (1:1000; Thermo Fisher/Invitrogen, cat. no. A-11008), goat anti-chicken IgY AlexaFluor 555 (1:1000; Thermo Fisher/Invitrogen, cat. no. A-21437), donkey anti-mouse IgG AlexaFluor 647 (1:1000; Thermo Fisher/Invitrogen, cat. no. A-31571). Hoechst 33342 (at 2 mg/ml; Millipore Sigma, cat. no. B2261) was used to mark nuclei, and coverslips were mounted on glass microscope slides using Fluoromount G (Fisher Scientific, cat. no. OB100-01). There were 2–3 coverslips per condition, and three separate trials were performed. Coverslips were stored at −20 °C between imaging sessions and were blinded during imaging and analysis.

For assessing the ratio of excitatory/inhibitory neurons, coverslips were imaged on a Zeiss LSM800 with AiryScan using a ×20 objective. Six images were taken per condition of wavelengths at 405, 488, 561, and 640 nm. Number of excitatory or inhibitory neurons were counted manually via the presence of VGLUT1 or GAD65/67, respectively. Co-localization of either synaptic marker with MAP2 was required to avoid counting dead neurons.

For assessing the ratio of excitatory/inhibitory synapses, the same coverslips were imaged on a PerkinElmer Spinning Disk microscope using a ×100 objective and a Hamamatsu ORCA-R2 CCD camera. Four z-stack images with a step size of 0.2 μm were taken per coverslip of wavelengths at 405, 488, 561, and 633 nm. Prior to automated analysis in MATLAB, z-stack images were maximum projected in FIJI. The 488 nm (GAD65/67), 561 nm (MAP2), and 633 nm (VGLUT1) channels were each analyzed separately. To segment inhibitory synapses (positive for GAD65/67), we use the following approach: (i) apply a Gaussian filter with a small sigma (*σ* = 1), (ii) binarize the image using an adaptive threshold (imbinarize in MATLAB with 'adaptive' option) to segment inhibitory synapses, (iii) apply an upper size threshold to eliminate false positive signals that are too large to be synapses. To segment excitatory synapses (positive for VGLUT1), we use the following approach: (i) remove noise by subtracting a Gaussian-filtered image (*σ* = 5) from the original image, (ii) apply a Gaussian filter with a small sigma (*σ* = 1), (iii) generate a binarized image where objects that are too bright or too dim relative to mean fluorescence have been eliminated, (iv) apply size thresholds to eliminate false positive signals that are too large or too small. As with the cell death analysis, to segment dendrites, MAP2 images were filtered using a rotating, two-dimensional Laplacian of Gaussian filter^73,74^. A binary image is generated from this filtered image, and a lower threshold is applied to eliminate small segments that are not true dendrites. Binarized dendrites are then dilated slightly. Any inhibitory (GAD65/67 positive) or excitatory (VGLUT1) synapses that overlap with dilated dendrites are counted.

### Preparation of microelectrode arrays (MEAs)

Standard 60-electrode MEAs (59 electrodes plus 1 reference electrode) were used for all experiments (60MEA200/10iR-Ti-gr, Multi-Channel Systems, Germany) and contain electrodes with diameters of 10 μm and inter-electrode spacings of 200 μm. MEAs were prepared for cell culture as we previously described^15–18^. Briefly, MEAs were washed for at least 48 hours in 1% Tergazyme (Fisher Scientific, cat. no. 16-000-115) solution (in dH_2_O) prior to the day of dissection. On the day of dissection, MEAs were autoclaved, rinsed once with sterile water, and left to dry in a sterile cell culture hood. MEAs were then coated with 0.5 mg/mL poly-d-lysine (PDL; MilliporeSigma, cat. no. P0899) and incubated at 37 °C for at least 1 hour. MEAs were then washed three times with sterile water and dried in a sterile cell culture hood. Immediately before plating of cells, MEAs were coated with 10 μg/ml laminin (MilliporeSigma, cat. no. L2020) for 30 min at 37 °C.

### MEA recordings

The spontaneous activity of hippocampal networks on MEAs was recorded using the data acquisition software MCRack (Multi Channel Systems, Germany, version 4.6.2). Recordings were performed at 37 °C on a heat-controlled stage at room atmosphere as previously described^15–18^. Data were acquired at a sampling rate of 20 kHz using an MEA1060-Inv-BC amplifier (Multi Channel Systems, Germany). A recording solution containing the following components was used to regularize bursting behavior (in mM): 144 NaCl 10 KCl, 1 MgCl_2_, 2 CaCl_2_, 10 HEPES, 2 Na-pyruvate, and 10 glucose at physiological pH (pH 7.4)^15–18^. During recording, MEAs were covered with semi-permeable lids (ALA MEA-MEM, Multi-Channel Systems) that selectively allow gases to diffuse through but that prevent airborne pathogens from contaminating the cultures. Before recording, cultures were equilibrated in the recording solution for 5-10 min. Spontaneous activity was then recorded for 5 min. Cultures were then washed once with growth medium, and treatment was applied or the conditioned medium was returned, as described below. MEA data was acquired through repeated recording of the same networks over time.

### Signal processing

All methods of MATLAB data analysis are based on our previous work^15–18^ but have been redeveloped specifically for the analysis of hippocampal neuron network activity. During recordings, electrodes showing excessive noise were noted and excluded from later analysis. Raw data were imported into MATLAB (MathWorks, Inc.) using MEAtools^75^, an open-source toolbox. Signals were filtered in 10 s chunks (300 s = 30 chunks total) through a fourth-order Butterworth bandpass filter (20–2000 Hz) and a 60 Hz notch filter to remove electrical noise. Importantly, both filters were infinite impulse response (IIR) filters and were implemented using the built-in MATLAB function *filtfilt()*, which is a zero-phase forward and reverse digital IIR filter. This type of filter does not introduce a time delay, unlike traditional finite impulse response (FIR) filters (the corresponding MATLAB function is *filter()*, a standard one-dimensional digital FIR filter).

### Spike detection and related parameters

Spikes are defined as single events in which the voltage surpasses a positive or negative threshold and are detected using an adaptive thresholding method. Spike thresholds were defined as 4.5 times the standard deviation of the background noise^76^ and were calculated for each 10 s period of the filtered signal. Importantly, the threshold is recalculated for each subsequent period and calculated separately for each electrode. The background noise can change over the recording period, and electrodes tend to have slightly different background noise levels. Spikes are detected at the maximum absolute value (positive or negative), and to ensure that the same spike is not counted twice, we require the interspike interval (ISI) to be at least 2 ms.

In addition to calculating spike rate (in spikes/s; Hz), we also calculate Fano factor, which measures the variability of the spike count within a specific window of time *w*. We determined this window *w* to be 100 ms for our in vitro networks. For 300 s recordings, we calculated the number of spikes that occurred in 100 ms bins (from msec 0–100, 101–200, etc.), giving us 3000 Fano factor count values per electrode. The Fano factor is calculated as the ratio of the variance to the mean of these spike counts^77^:

1 $${{\rm {FF}}}={\sigma }_{{\rm {w}}}^{2}\,({{\rm {spike}}}\,{{\rm {count}}})/{\mu }_{{\rm {w}}}\,({{\rm {spike}}}\,{{\rm {count}}})$$

Since the majority of the spiking activity in our networks is present within burstlets, we used a window *w* that corresponds to the same order of magnitude as the length of our burstlets. The Fano factor tends toward 0 for networks with regularly spaced spikes^78,79^. When the spike rate is random and follows a Poisson distribution, the Fano factor is theoretically equal to 1^77,80^. Higher Fano factor values are indicative of irregular firing, which is observed in our hippocampal neuron networks^17,18,78,79^.

### Burstlet detection

Spikes occurring in rapid succession on an electrode are referred to as an individual bursts or “burstlet”. After all spikes are detected, we determine whether spikes are part of a burstlet, which are event composed of a core group of very closely spaced spikes and a peripheral group of less closely spaced spikes. To implement these criteria for the detection of burstlets, our algorithm searched for groups of at least 4 spikes with ISIs of 100 ms or 4 times the firing rate of that electrode, whichever was smaller. Upon finding the core groups of spikes, the algorithm searched for peripheral spikes that had ISIs of 200 ms or 3 times the firing rate of that electrode, whichever was smaller^76^. After all burstlets in each 10 s period were detected, the algorithm checked whether, on any electrode_*i*_, a burstlet at the end of period_*j*_ overlapped with a burstlet at the beginning of period_*j*+1_. If so, these burstlets were combined into one.

### Global burst detection

Our hippocampal networks not only exhibit random spiking and bursting activity on individual electrodes, but they will also often display network-wide bursts, known as synchronized bursting events^81^, which occur when multiple electrodes record burstlets that overlap in time. Physiologically, synchronized bursting events represent the synchronous activity that is necessary for many brain functions^82,83^, and thus, are an important measure of network activity. Here, we refer to these network-wide bursts as global bursts, and they are detected as at least three overlapping burstlets on different electrodes^76^.

### BDNF treatment of developing hippocampal networks and recording schedule

Cultures of hippocampal neurons were maintained for 7 days in vitro (DIV) prior to treatments and recordings. The timeline used for these experiments and abbreviations used in the text and figures are shown in Supplementary Fig. 2a.

Baseline recordings were performed at DIV 7 using MCRack software. Immediately after recording, an activity check was performed. Cultures with <2000 spikes in 5 min (a spike rate of <6.7 Hz) were not used for further experimentation. Remaining cultures that did pass the activity threshold were randomly assigned to one of three treatment groups: control (0 ng/ml BDNF; 0B), 25 ng/ml BDNF (25B), or 50 ng/ml BDNF (50B). BDNF or vehicle (sterile water) was added to the conditioned medium, which was then applied to cultures for 72 hours. We used the additional treatment concentration of 50 ng/ml because MEA cultures are plated 3.5 times more densely than cultures used in previous work studying BDNF-mediated effects on dendrite morphology^27,29,30^. At DIV 10, after 72 hours of BDNF or vehicle treatment, an additional recording was performed, and the treatment medium was replaced with a regular culture medium. Importantly, this treatment window corresponds to that of our previous work, in which BDNF was applied to cultures of hippocampal neurons for 72 hours, resulting in increases in proximal branching^27^. A final recording was performed at DIV 17, which is 7 days post-treatment, to determine whether BDNF exerts any long-term effects on network dynamics.

### Identification of glutamate concentration that results in sublethal injury of hippocampal networks

To determine the concentration of glutamate that results in a mild injury of hippocampal networks, cultures were maintained for 14 DIV prior to injury with glutamate and recording. The timeline used for these experiments is shown in Supplementary Fig. 2b.

Baseline recordings were performed at DIV 14 using MCRack software. Immediately after recording, an activity check was performed using the aforementioned criteria. Cultures that passed the activity threshold were randomly assigned to one of five injury groups: no injury (0 μM glutamate; 0g), 30 μM glutamate (30g), 100 μM glutamate (100g), 175 μM glutamate (175g), or 250 μM glutamate (250g). Glutamate or vehicle (sterile water) was added to the conditioned medium, which was then applied to cultures for the following amount of time: injury with higher levels of glutamate (175 and 250 μM) was induced for 1 hour^15,16^, while injury with lower levels of glutamate (30 and 100 μM) was induced for 30 min. After injury, the glutamate-containing medium was replaced with a conditioned medium.

Our goal for studying glutamate-induced excitotoxicity was to cause decreases in activity but not total elimination of activity, and thus, we quantified how the different concentrations of glutamate affected spike rate (Supplementary Fig. 2c1), burstlet rate (Supplementary Fig. 2c2), and global burst rate (Supplementary Fig. 2c3). Injury with higher concentrations of glutamate (100g, 175g, and 250g) results in significant decreases for all activity metrics both compared to the control (# symbols) and compared to their raw values pre-injury (* symbols). In contrast, injury with the lowest concentration of glutamate (30g) results in significant decreases in spike rate, burstlet rate, and global burst rate compared to the control (# symbols), but only global burst rate decreased compared to pre-injury levels (* symbol). Thus, we selected 30 μM as our excitotoxic injury.

### Injury of hippocampal networks with glutamate and recovery treatment with BDNF

After determining the concentration of glutamate that resulted in sublethal injury to networks of hippocampal neurons (30 μM glutamate for 30 min), cultures were maintained for 14 DIV before treatments and recordings. The timeline used for these experiments and abbreviations used in the text and figures are shown in Supplementary Fig. 2d.

Baseline recordings were performed at DIV 14 using MCRack software. Immediately after recording, an activity check was performed using the aforementioned criteria. Cultures that passed the activity threshold were randomly assigned to one of two injury groups: no injury (0 μM glutamate; 0g) or 30 μM glutamate (30g). Glutamate or vehicle (sterile water) was added to conditioned medium, which was then applied to cultures for 30 min. After injury, cultures were randomly assigned to one of two treatment groups: no treatment (0 ng/mL BDNF; 0B) or treatment with 50 ng/mL BDNF (50B). Cultures were maintained in treatment medium with or without BDNF until the 24h post-injury recording on DIV 15. After this recording, the treatment medium with or without BDNF was reapplied, and cultures were maintained in this medium until the 72h post-injury recording on DIV 17.

### Synchronization calculation

Synchronization between electrodes is based on the overlapping of individual burstlets on different electrodes and is referred to as synchrony of firing (SF). This type of synchronization measure indicates how correlated the bursting of one electrode is with other electrodes. SF is calculated by taking the ratio of the number of times electrodes *x* and *y* burst together (*B*_*x*&*y*_) versus the maximum number of times either electrode bursts on its own (*B*_*x|y*_)^15–18^

2 $${{\rm {SF}}}={B}_{x{{\& }}y}/{B}_{x{{{{{\rm{|}}}}}}y}$$

Here, a value of 0 indicates no synchronization and a value of 1 indicates full synchronization. To assess changes in synchronization, we examined the changes that occur to electrodes possessing specific baseline levels of synchronization. As in our previous work, we categorized electrodes into the following initial synchronization bins (categories): 0.1–0.4 (weak), 0.4–0.7 (medium), and 0.7–1.0 (strong)^15,16^. We ignored electrodes with initial synchronizations of between 0 (no synchronization) and <0.1 (very weak synchronization) to prevent large percent changes from biasing the results.

### Network parameters: local efficiency

To examine how BDNF treatment and glutamate injury affected the functional connectivity of networks, we used the Brain Connectivity Toolbox (BCT; brain-connectivity-toolbox.net;^84^) to calculate the local efficiency for both in vitro and in silico networks. Local efficiency is the average inverse shortest path length of a node’s neighbors and measures the resilience of the network to injury on a local scale. It is a measure of segregation and is calculated for each of the 59 active electrodes on the 60-electrode MEA (59 active electrodes +1 reference electrode, as in our previous work^15,16^). To measure local efficiency, we first generate functional connectivity matrices by binning spikes and calculating cross-correlation, as in our previous work^17^. These functional connectivity matrices are undirected network matrices with a size of 59 × 59 that are then inputted into BCT. Our binning parameters are 10 ms for in vitro datasets and 1 ms for in silico datasets. The slight difference in parameter choice arises from the lower amount of chatter and a higher level of burstiness in the in silico networks compared to in vitro networks. The binning parameter used for in silico datasets is consistent with our previous study^17^. Since our in vitro networks in this study showed significantly higher levels of burstiness than the in vitro networks in our previous work^17^, it was necessary to choose a larger binning window to account for bursting behavior. Note that the undirected network matrices used for local efficiency analysis are distinct from the directed networks used for Granger causal analysis (described below in subsection “Estimation of directed network structure using Granger causality analysis”).

### Tracking electrode data over time

In contrast with our previous studies, we chose in this work to track electrodes over time to better understand how hippocampal networks change during development and after injury. After calculating parameters for each electrode, we only kept track of electrodes that functioned properly (i.e., not too noisy) for all recording periods. For synchronization categorization, the initial category is determined by the binning on the first day of recording (DIV 7 or 14).

### Extraction of burstlet trains for Granger causality and higher-order synchrony analysis

We observed that spiking activity in each electrode of the MEA recordings occurred primarily in burstlets. Defining a burstlet to be a 300 ms period during which at least 4 spikes are observed, we formed binary time series by binning spike times at a resolution of 300 ms and assigning bins with at least four spikes a value 1, and 0 otherwise. We used the burstlet trains in our statistical approaches for analyzing network-level properties of hippocampal neurons cultured on MEAs.

### Estimation of directed network structure using Granger causality analysis

To examine networks of directed interactions between active electrodes on the MEA, we use Granger causality (GC) analysis, which tests if the bursting activity of one electrode is better predicted by knowledge of the recent bursting history of a second electrode. Recent work^24,25^ developed a GC measure for point processes that accounts for the sparsity of interactions and controls the false discovery rate (FDR) of GC links. We adapted the point process GC framework to infer functional interactions between active electrodes using extracted burstlet trains. Then, using the total number of links in GC networks as a measure of network connectivity, we compared the mean change across experimental conditions and days in vitro to identify significant changes, as determined by the two-sided Wilcoxon rank sum test. The following subsections formulate the point process likelihood model and describe the estimation and statistical inference procedure for GC analysis.

#### Point process likelihood model for burstlet trains

Suppose $C$ active electrodes were recorded. The observed burstlet sequence of the ${c}{{{{{{\mathrm{th}}}}}}}$ unit $\{{n}_{t}^{\left(c\right)}\}_{t=1}^{T}$ is treated as a sequence of Bernoulli random variables whose success probabilities are given by the conditional intensity function (CIF) $\{{\lambda }_{t}^{(c)}\}_{t=1}^{T}$. The CIF is modeled by a generalized linear model (GLM) with logistic link function:

3 $${\lambda }_{t}^{(c)}=\frac{\exp \left({{{{{{{\boldsymbol{\omega }}}}}}}^{\left(c\right)}}^{{\prime} }{{{{{{\boldsymbol{x}}}}}}}_{t}\right)}{1+\exp \left({{{{{{{\boldsymbol{\omega }}}}}}}^{\left(c\right)}}^{{\prime} }{{{{{{\boldsymbol{x}}}}}}}_{t}\right)}$$

The parameter vectors consist of a baseline firing rate parameter and history modulation vectors for each active electrode, i.e., ${{{{{{\boldsymbol{\omega }}}}}}}^{(c)}={[{\mu }^{(c)},{{{{{{{\boldsymbol{\omega }}}}}}}^{(c,1)}}^{{\prime} },\ldots ,{{{{{{{\boldsymbol{\omega }}}}}}}^{(c,C)}}^{{\prime} }]}^{{\prime} }$; and the history covariate vector at time $t$ consists of the recent burst history of each electrode, ${{{{{{\boldsymbol{x}}}}}}}_{t}=[1,{{{{{{{\boldsymbol{n}}}}}}}_{t}^{(1)}}^{{\prime} },\ldots ,{{{{{{{\boldsymbol{n}}}}}}}_{t}^{(C)}}^{{\prime} }]$.

The combined log-likelihood of the sequence of observations is expressed as the sum of Bernoulli log-likelihoods over time:

4 $${{{{{\mathscr{L}}}}}}({{{{{{\boldsymbol{\omega }}}}}}}^{(c)})=\mathop{\sum }\limits_{t=1}^{T}{{{{{{\boldsymbol{n}}}}}}}_{t}^{(c)}{{{{{{{\boldsymbol{\omega }}}}}}}^{(c)}}^{{\prime} }{{{{{{\boldsymbol{x}}}}}}}_{t}-\log \left(1+\exp \left({{{{{{{\boldsymbol{\omega }}}}}}}^{\left(c\right)}}^{{\prime} }{{{{{{\boldsymbol{x}}}}}}}_{t}\right)\right)$$

#### Model estimation and statistical inference of GC links

To determine if a GC link from a source electrode $\bar{c}$ to a target electrode $c$ exists, two-point process models of the target’s burstlet activity are estimated and compared: a *full* model that includes the recent burstlet activity history of all active electrodes as covariates; and a *reduced* model, nested within the *full* model, that excludes the activity history of the source electrode from the covariates.

The full model is obtained by solving

5 $${\hat{{{{{{\boldsymbol{\omega }}}}}}}}^{(c)}=\mathop{{{{{{\rm{argmax}}}}}}}\limits_{{{{{{{\boldsymbol{\omega }}}}}}}^{(c)}}{{{{{\mathscr{L}}}}}}\left({{{{{{\boldsymbol{\omega }}}}}}}^{\left(c\right)}\right)$$

using a generalized orthogonal matching pursuit (OMP)^85,86^ to enforce parameter sparsity. OMP iteratively updates and optimizes over the model support set, or the subset of non-zero parameters of ${\hat{{{{{{\boldsymbol{\omega }}}}}}}}^{(c)}$, by first identifying the parameter for which the partial gradient of the log-likelihood has the largest magnitude and then solving the maximum-likelihood problem over the updated support. The sparsity level (support size) is determined by cross-validation, hence accounting for the possibility that the interactions may be dense.

The reduced model is estimated similarly by solving the maximization problem

6 $${\hat{{{{{{\boldsymbol{\omega }}}}}}}}^{(c{{{{{\rm{\backslash }}}}}}\bar{c})}=\mathop{{{{{{\rm{argmax}}}}}}}\limits_{{{{{{{\boldsymbol{\omega }}}}}}}^{(c{{{{{\rm{\backslash }}}}}}\bar{c})}}{{{{{\mathscr{L}}}}}}({{{{{{\boldsymbol{\omega }}}}}}}^{(c{{{{{\rm{\backslash }}}}}}\bar{c})})$$

where the likelihood is evaluated with the reduced set of history covariates, ${{{{{{{\boldsymbol{x}}}}}}}^{{{{{{\boldsymbol{\backslash }}}}}}\bar{{{{{{\boldsymbol{c}}}}}}}}}_{t}$, that exclude the history of the ${\bar{c}}{{{{{{\mathrm{th}}}}}}}$ unit.

A GC link from the source to the target electrode is detected if the inclusion of the recent activity history of the source electrode significantly improves the prediction of the target electrode’s activity. That is, if the likelihood of the full model is significantly greater than that of the reduced model, the null hypothesis that there is no GC link is rejected. Hence, the predictivity of the full and reduced models are compared using the deviance difference

7 $${D}^{(\bar{c}\mapsto c)}=2\left[{{{{{\mathscr{L}}}}}}\left({\hat{{{{{{\boldsymbol{\omega }}}}}}}}^{\left(c\right)}\right){{{{{\mathscr{-}}}}}}{{{{{\mathscr{L}}}}}}\left({\hat{{{{{{\boldsymbol{\omega }}}}}}}}^{\left(c{{{{{\rm{\backslash }}}}}}\bar{c}\right)}\right)\right]$$

a statistic frequently used for likelihood ratio tests with nested hypotheses. Since the full model has more degrees of freedom than the reduced model, the deviance difference is non-negative with large values indicating potential GC links. Using the inference framework in recent work^24,25^, which precisely characterizes the distribution of the deviance difference, we test the significance of the deviance difference while controlling the false discovery rate at $\alpha =0.01$ using the Benjamini–Hochberg procedure^87^. Repeating this procedure for all pairs of source and target electrodes $(\bar{c},c)$ yields the GC network.

### Higher-order synchrony analysis

We characterized network synchrony in further detail by adapting a recently developed modeling and inference framework for single-trial spiking data^35,36^ to burstlet activity. Specifically, we used a static history-independent model of ensemble spiking to test whether $r$-wise simultaneous bursting occurs at a significantly higher rate than expected from independent electrodes, as summarized here.

In contrast to the conditionally independent set of point process models for each unit used in GC analysis, we used a marked point process (MkPP) model that jointly characterizes all permutations of ensemble bursting. That is, for $C$ electrodes, each of the ${C}^{* }={2}^{C}-1$ ensemble states (or marks), ${{{{{{\boldsymbol{n}}}}}}}_{t}={[{n}_{t}^{(1)},{n}_{t}^{(2)},\ldots ,{n}_{t}^{(C)}]}^{{\prime} }$, is represented by categorical vectors ${{{{{{\boldsymbol{n}}}}}}}_{t}^{* }={[{{n}_{t}^{* }}^{(1)},{{n}_{t}^{* }}^{(2)},\ldots ,{{n}_{t}^{* }}^{({C}^{* })}]}^{{\prime} }$. Hence, all permutations of ensemble bursting are represented disjointly so that their rates may be modeled directly in the joint log-likelihood

8 $$\log p\left({{{{{{\boldsymbol{n}}}}}}}_{t}^{* }\right)={{{{{{\boldsymbol{\mu }}}}}}}^{{\prime} }{{{{{{\boldsymbol{n}}}}}}}_{t}^{* }-\psi \left({{{{{\boldsymbol{\mu }}}}}}\right),\psi \left({{{{{\boldsymbol{\mu }}}}}}\right)=\log \left(1+\mathop{\sum }\limits_{m=1}^{{C}^{* }}{{\rm {e}}}^{{\mu }^{\left(m\right)}}\right)$$

where ${{{{{\boldsymbol{\mu }}}}}}$ is the rate parameter vector and $\psi ({{{{{\boldsymbol{\mu }}}}}})$ is a normalization factor.

To test for ${r}{{{{{{\mathrm{th}}}}}}}$-order synchrony (i.e., a significantly high rate of $r$-wise simultaneous bursting), we estimated two nested MkPP models. The full model parameters are estimated without constraint while the reduced model parameters are estimated with the rate parameters of ${r}{{{{{{\mathrm{th}}}}}}}$-order marks fixed at the values expected if ${r}{{{{{{\mathrm{th}}}}}}}$-order events occurred by chance. In a similar manner as in the inference of GC links, the full and reduced models are compared using the deviance difference; ${r}{{{{{{\mathrm{th}}}}}}}$-order synchrony is detected if the deviance difference is significantly greater than zero. The analysis is repeated for each $r=2,\ldots ,C$ and thus, characterizes all higher-order synchronous bursting across the MEA. For the sake of numerical tractability, only electrodes with significant activity above a threshold of 30 burstlets were retained in the analysis of higher-order synchrony.

### Visualizing spatial distributions of synchronous units

To visualize the localization of synchronous electrodes, we generated histograms of the distance between each electrode and its midpoint. For example, suppose a set of $r$ synchronous electrodes have positions $({x}_{i},{y}_{i}),{i}=1,\ldots ,r$; we compute the distance vectors $({x}_{i}-\bar{x},{y}_{i}-\bar{y}),i=1,\ldots ,r$, where $\bar{x}=\mathop{\sum }\nolimits_{i=1}^{r}{x}_{i}$ and $\bar{y}=\mathop{\sum }\nolimits_{i=1}^{r}{y}_{i}$. These distance vectors are compiled separately for low (2–8), intermediate (9–15), and high (16+) orders of synchrony and displayed as a spatial histogram. Larger counts in the center of the spatial histogram indicate more localized synchronous units. The similarity of pairs of spatial distributions was quantitatively determined by two-sample KS tests (significance level *p* < 0.05).

### Tokeshi’s test of bimodality

To assess differences in the frequency distribution of orders of synchrony, we adapted Tokeshi’s test for bimodality^37^. The test was developed to distinguish types of modality in spatial frequency distributions of animal communities but is applied to our setting to analyze the modality of frequency distributions of present orders of synchrony.

First, we bin the orders of synchrony. This reduces the number of synchrony classes and highlights peaks in the distribution, similar to the use of a larger class interval in previous work^37^. Two candidate modes are selected by identifying one peak amongst the lower- and higher-order synchrony classes each. The selected classes are, respectively denoted, by ${m}_{{\rm {l}}}$ (${m}_{{\rm {r}}}$) with frequencies ${n}_{{\rm {l}}}$ (${n}_{\rm {{r}}}$). Three probabilities are computed under the null hypothesis: the sum of probabilities of obtaining at least the observed frequencies ${n}_{\rm {{l}}}$ and ${n}_{{\rm {r}}}$, ${P}_{\rm {{c}}}$; and, separately, the probability of obtaining at least ${n}_{{\rm {l}}}$ (${n}_{{\rm {r}}}$), ${P}_{\rm {{l}}}$ (${P}_{\rm {{r}}}$). Explicitly, with $N$ the total number of samples and $\frac{1}{h}$ the number of classes,

9 $${P}_{{\rm {c}}}=\mathop{\sum }\limits_{i={n}_{{\rm {l}}}}^{N-{n}_{{\rm {l}}}}\mathop{\sum }\limits_{j={n}_{{\rm {r}}}}^{N-i}\frac{N!{h}^{i+j}{(1-2h)}^{N-i-j}}{i!j!(N-i-j)!}$$

10 $${P}_{l}=\mathop{\sum }\limits_{i={n}_{l}}^{N}\left({N}\atop{i}\right){h}^{i}{(1-h)}^{N-i}$$

and

11 $${P}_{r}=\mathop{\sum }\limits_{i={n}_{r}}^{N}\left({N}\atop{i}\right){h}^{i}{(1-h)}^{N-i}$$

We also define an additional quantity $\rho (t)={[1-\max ({P}_{{\rm {l}}},{P}_{{\rm {r}}})]}^{t}$ that is used to reflect the relative magnitude of a small peak. If *t* consecutive classes adjacent to the class (${m}_{{\rm {l}}}$ or ${m}_{\rm {{r}}}$) that maximizes $[\max ({P}_{{\rm {l}}},{P}_{{\rm {r}}})]$ have frequencies lower than ${n}_{\rm {{l}}}$ (or ${n}_{\rm {{r}}}$) and $t$ satisfies $\rho (t) < 0.1$, that class is judged to be locally significant. Table 1 summarizes Tokeshi’s test of bimodality based on the values of ${P}_{\rm {{c}}}$, ${P}_{{\rm {l}}}$, ${P}_{{\rm {r}}}$, and $\rho (t)$.

### Computational neuron network model

For our simulations, we modified a neuron network model developed in Python 3.8 that uses the Brian2 neural simulator^41,88^. Our group has previously adapted this model to replicate and extend in vitro findings^17^. In this work, our network employs distance-dependent connections^89^ that we then adjusted in accordance with our previously reported in vitro data^27^. Our network contains 500 neurons seeded at a density of 3.5 × 10^3^ cells/mm^2^ to match in vitro cell density and comprises 440 excitatory neurons and 60 inhibitory neurons (E/I balance of 88/12) to match in vitro E/I neuronal balance (Fig. 5d, e). We connected our neurons probabilistically based on the neuron-neuron distance and type of connection based on Voges and Perrinet (2012)^89^ and scaled the likelihood function to achieve the target E/I synaptic balance of ~65/35 to match in vitro E/I synaptic balance (Supplementary Fig. 8 for in silico; Fig. 5f and Supplementary Fig. 5 for in vitro).

We used a conductance-based leaky integrate and fire model for our neurons. Their membrane potential *V* follows the Langevin equation:

12 $${{C}_{{\rm {m}}}\frac{{{\rm {d}}V}}{{{\rm {d}}t}}=-{g}_{{\rm {m}}}\left(V-{V}_{{\rm {L}}}\right)-{I}_{{{\rm {syn}}}}+{I}_{{{\rm {AHP}}}}+I}_{{{\rm {pre}}}}$$

where *C*_m_ is the capacitance, *g*_m_ = *1/R*_m_ is the membrane leak conductance, and *V*_L_ is the resting potential. The synaptic current *I*_syn_ represents the sum of the glutamatergic AMPA and NMDA excitatory currents and GABA inhibitory currents. The after-hyperpolarization current *I*_AHP_ represents slow calcium dynamics and fatigue. *I*_pre_ represents presynaptic neuronal noise current, modeled as Gaussian white noise. Excitatory–excitatory connections are modified by spike-time-dependent plasticity^90^. The excitatory–excitatory synapses are initially seeded at a weight of 8 AU and capped at 16 AU to prevent runaway bursting. All other synapses have a weight of 1. Only one synapse can exist between a given set of two neurons.

We seeded our neurons with a Gaussian distribution of external current inputs and allowed the network to stabilize for 120 s, after which period the spike and burst rate experienced only marginal changes in unperturbed networks. We ran each simulation 6 times, consistent with the replicates of in vitro recordings.

The code used to generate our neuron network model can be found on the Meaney Lab website (https://www.seas.upenn.edu/~molneuro/software.html) and ModelDB.

### Glutamate and BDNF simulation in silico

To simulate the application of glutamate in silico, we replicated the findings in vitro with regard to loss of excitatory and inhibitory neurons and synapses (Fig. 5 and Supplementary Fig. 5). We intended for “Injury” to represent 30 µM glutamate and for “post” to represent the 72h post timepoint. We silenced 30% of excitatory neurons, 25% of inhibitory neurons, and 75% of inhibitory synapses. To preserve bursting activity and account for the low plating density required for imaging studies to assess cell death, we proportionally scaled back the cell death applied in silico. Owing to the inhibitory synaptic loss associated with inhibitory cell death, this resulted in a net loss of inhibitory synapses of 86% in our model (Supplementary Fig. 8c, d).

To simulate the application of BDNF in silico, we again replicated the findings in vitro with regard to the protection of inhibitory neurons (Fig. 5e). We intended our “BDNF treatment” to correspond to treatment with 50 ng/ml BDNF. We modeled BDNF as a reintroduction of 50% of injured inhibitory neurons after the network settled from simulated glutamate injury (net reintroduction of 7 inhibitory neurons; Supplementary Fig. 7). Reintroduced neurons had the same connections as they did pre-injury unless their connections were specifically injured as a result of glutamate injury (Supplementary Fig. 8a). Since we modeled BDNF as a resurrection of lost neurons, we only modeled BDNF treatment in the post-injury case and not as a control treatment.

To replicate the timing of BDNF and glutamate treatment in vitro (Fig. 5a), we first allowed the in silico network to settle from 0 to 120 s (Fig. 9a; as described in the section “Methods”*:* subsection “Computational neuron network model”). We then allowed the simulation to run from 120 to 240 s to establish a “pre-treatment” period against which the effects of treatment(s) would be compared (Fig. 9a). The pre-treatment period was matched for each set of Control, Injury, and Injury + BDNF simulations to minimize the effect of variable network parameters (e.g., neuron location and its effect on connectivity) on treatment effect. Glutamate was always applied at the beginning of the first treatment epoch (at 240 s), and the network was allowed to resettle for 120 s. If BDNF was applied, it occurred at the beginning of the second treatment epoch (at 360 s), and the network was allowed to settle for 120 s. If BDNF was not applied, the network was allowed to run unperturbed for 120 s during the second treatment epoch. Next, the simulations entered the “post-treatment” epoch from 480 to 600 s: the period when we evaluated the effect of treatment(s).

### Conversion of neuron network model to MEA-type recording

Because of the significant cell loss intrinsic to this model of neuronal injury, we converted our in silico neuronal recordings into a pseudo-MEA recording to be more faithful to the in vitro experimental design. MEAs in vitro are understood to record from approximately 3–6 neurons^15–18^. In accordance, we spatially divided our 500 neurons into an 8 × 8 grid and then removed 5 reference electrodes to equal the 59 recording electrodes found in vitro (Supplementary Fig. 7). As a result, each “electrode” in our system records from an average of 7.8 neurons in the uninjured condition (Supplementary Fig. 7d).

After consolidating our in silico neuron-based recordings to MEA-like recordings, we analyzed the simulations in the same manner as the in vitro MEA recordings, evaluating burstlet rate, Fano factor, and local efficiency.

### Data representation

For all MEA-derived parameters, data are represented as percent change and compared to the baseline timepoint. In cases where a particular parameter can be tracked for each electrode (e.g., burstlet rate), each datapoint represents an electrode value. For these parameters, we only used datasets that included electrodes that could be tracked across all three timepoints. To prevent artificial inflation of percent change values for in vitro and in silico data, the following thresholds (per electrode) were used: 0.2 Hz for spike rate, 0.02 Hz for burstlet rate, 0.01 Hz for global burst rate, and 0.005 (AU) for local efficiency. It was not necessary to use a lower threshold for Fano factor, and the synchronization data already have a lower bound of 0.1.

All data were plotted using MATLAB 2021a. Error bars indicate 95% CIs and are occasionally smaller than the size of the mean datapoint when the number of datapoints is large. We used Adobe Illustrator (version 25.0.1) for the final assembly of figures.

### Statistics and reproducibility

In cases where a particular parameter must be calculated as one value for each MEA network (e.g., Granger Causal links), then each datapoint represents an MEA network value. For these parameters, we used all available data. In the figure legends, we specify that *N* indicates independent experiments, *e* indicates electrodes, and *n* indicates datapoints (MEA networks). For some in silico data, we also report that *s* indicates a number of synapses.

In rare cases, we excluded data using the median absolute deviation (MAD) calculation. We used a scale factor *k* = 2 for the Western blot analysis (Fig. 1c; eliminated one experiment) and preliminary glutamate experiments (Supplementary Fig. 2c; eliminated two datapoints from the control condition for all parameters). It was also necessary to use this outlier removal method for the final normalized data from the in silico networks (scale factor *k* = 3).

When tracking electrode data over time, we used repeated measures ANOVA to determine significant differences between timepoints within the same condition. To keep RM ANOVA as accessible as possible for readers, we treat the conditions in the injury dataset as one set of four categories (untreated uninjured, treated uninjured, untreated injured, treated injured) rather than two sets of two categories (treated/untreated, injured/uninjured). We report F-statistics and *p* values for all parameters analyzed by RM ANOVA in Supplementary Tables 1, 2, and 3. After RM ANOVA, we use Tukey–Kramer as a multiple comparisons test based on the treatment group for each timepoint separately. These data and statistical analyses are represented in plots with dual axes. Plots on the left side (“changes within”) demonstrate differences within a condition across timepoints (e.g., 24h post-injury versus pre-injury), and significance is shown via asterisks. For determining significant differences in changes across conditions, we used estimation statistics to calculate confidence intervals (CIs)^34^ (nIterations = 500) and then calculated the *p*-value from the CIs^91^. These data and analyses are shown on the right side of plots (“differences between”) and demonstrate how changes differ across treatment conditions. Statistical differences between the two conditions are shown by *p* values.

For E/I neuron data, we used Kruskal–Wallis followed by the Tukey–Kramer multiple comparisons tests (Fig. 5d, e). For E/I synapse data and, we used one-way ANOVA followed by Tukey–Kramer multiple comparisons test (Fig. 5f and Supplementary Fig. 5b–g). Statistical comparisons of the number of GC links across timepoints and conditions were made using a two-sided Wilcoxon’s rank sum test (Figs. 4a and 8a), while bimodality of the frequency distributions of higher-order synchrony was determined by Tokeshi’s test (Fig. 4b, c; see also Table 1). Differences in spatial distributions of synchronous units were tested using two-sample Kolmogorov–Smirnov (KS) tests (Figs. 4d and 8b, c). Changes to excitatory–excitatory synaptic weights for in silico networks were assessed via two-sample Kolmogorov–Smirnov test (Fig. 9g) and one-way ANOVA followed by Tukey–Kramer multiple comparisons test (Fig. 9h).

All statistical tests were performed using MATLAB 2021a. When datapoints are data from individual electrodes, individual data points are shown in the Supplementary Information along with the mean. At least three separate replicates (independent experiments) were performed for all datasets; we consider different dissections to be separate replicates. The significance level was set at *α* = 0.05.

### Reporting summary

Further information on research design is available in the Nature Portfolio Reporting Summary linked to this article.

### Supplementary information

Supplementary Information

Reporting summary

## Data Availability

The source data for all plots is publicly available on Figshare^92^.

## Code availability

All code is available on GitHub.com and on ModelDB.com. Refer to GitHub and the associated DOI for the MEA analysis code (github.com/katemon/MEAanalysis_ONeill2023/)^93^. Refer to GitHub and the associated DOI for the Granger causality analysis code (github.com/ShoutikM/GCandSynchronyAnalysisCode_ONeill_etal_NatCommunBiol_2023/)^94^. Please refer to ModelDB for the simulation code (https://modeldb.science/2015421).
