# Supplementary material for: Time-dependent homeostatic mechanisms underlie brain-derived neurotrophic factor action on neural circuitry
Source: Commun Biol. 2023 Dec 18;6:1278. doi: 10.1038/s42003-023-05638-9 (PMC10728104; doi:10.1038/s42003-023-05638-9)
Supplement: Supplementary file 1 — Supplementary Information [file 42003_2023_5638_MOESM1_ESM.pdf]

Supplementary Materials for

Time-dependent homeostatic mechanisms underlie Brain-Derived Neurotrophic Factor action on neural circuitry

Kate M. O'Neill, Erin D. Anderson, Shoutik Mukherjee, Srinivasa Gandu, Sara A. McEwan, Anton Omelchenko, Ana R. Rodriguez, Wolfgang Losert, David. F. Meaney, Behtash Babadi, Bonnie L. Firestein *

*Corresponding author. Email: firestein@biology.rutgers.edu

This PDF file includes the following:

Supplementary Text
Supplementary Figures 1-9
Supplementary Tables 1-3

SUPPLEMENTARY TEXT

Supplementary statistical details for Figure legends:

Statistics for Figure 4d: Based on two-sample KS tests, the following statistically significant differences in spatial distributions were observed. 0B: low-order pre vs. intermediate-order pre ($p<0.001$), low-order pre vs. high-order pre ($p<0.001$). 25B: low-order pre vs. high-order pre ($p<0.001$). 50B: low-order pre vs. intermediate-order pre ($p<0.001$), low-order pre vs. high-order pre ($p<0.001$). For low-order (D1): 0B: 0d post vs. 7d post ($p=0.0186$); 50B: pre vs. 0d post. For intermediate-order (D2): 0B: pre vs. 0d post ($p<0.001$), 0d post vs. 7d post ($p=0.013$); 25B: pre vs. 0d post ($p=0.044$), 0d post vs. 7d post ($p=0.001$); 50B: pre vs. 0d post ($p=0.011$), 0d post vs. 7d post ($p<0.001$).

Statistics for Figure 8b-c: Based on two-sample KS tests, the following statistically significant differences in spatial distributions were observed. For 0B low-order (b2): 0g0B: pre vs. 24h post ($p<0.029$), 24h post vs. 72h post ($p=0.005$); 30g0B: pre vs. 24h post ($p<0.001$). For 0B intermediate-order (b3): 0g0B: pre vs. 24h post ($p<0.001$). For 50B intermediate-order (c2): 0g50B: pre vs. 24h post ($p=0.008$), 24h post vs. 72h post ($p=0.032$).

SUPPLEMENTARY FIGURES

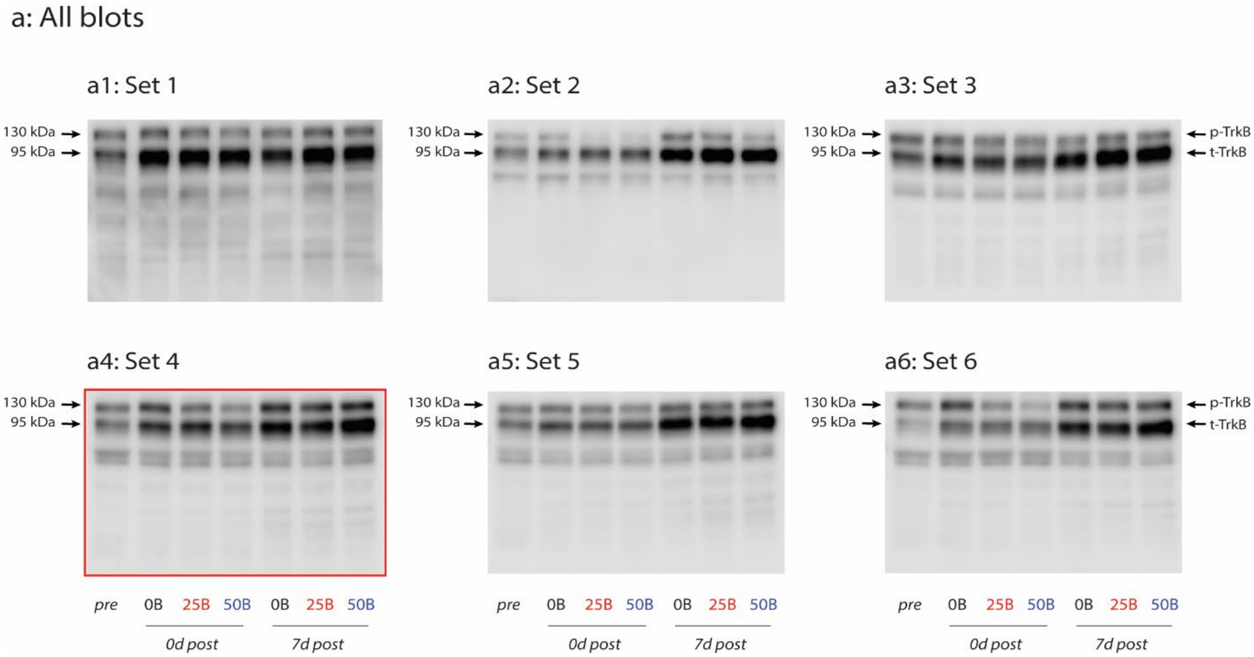

Supplementary Figure 1: All blots showing changes in ratio of phosphorylated (p-TrkB) to total TrkB (t-TrkB) as a result of BDNF treatment. All blots with p-TrkB (~140 kDa) and t-TrkB (~90 kDa) bands marked. Red box indicates representative blot from Fig. 1b. N=6 independent experiments.

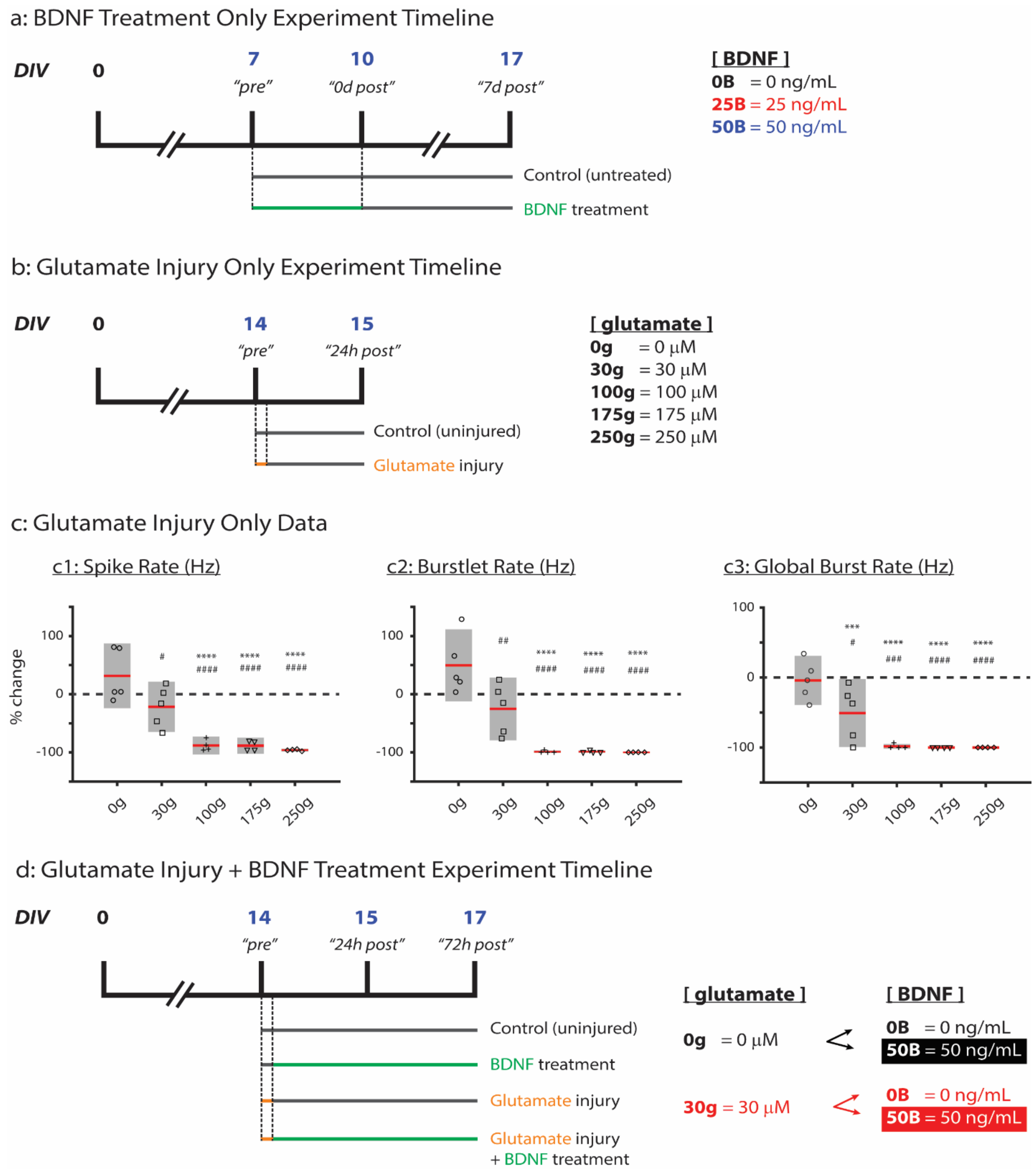

Supplementary Figure 2: Experimental timelines and pilot data for determining extent of glutamate damage on hippocampal networks. a) Timeline for BDNF dose response experiments. **b)** Timeline for pilot experiments to determine concentration for glutamate injury. **c)** Pilot data used to determine which concentrations of glutamate to choose for sublethal injury. **c1)** Spike rate significantly decreases at 24 hours after injury compared to pre-injury levels (* symbols) for 100g, 175g, and 250g and compared to the control (# symbols) for 30g, 100g,

175g, and 250g. **c2)** Burstlet rate significantly decreases at 24 hours after injury compared to pre-injury levels (* symbols) for 100g, 175g, and 250g and compared to the control (# symbols) for 30g, 100g, 175g, and 250g. **c3)** Global burst rate significantly decreases at 24 hours after injury compared to pre-injury levels (* symbols) for 30g, 100g, 175g, and 250g and compared to the control (# symbols) for 30g, 100g, 175g, and 250g. 0g = no glutamate; 30g = 30 μM glutamate; 100g = 100 μM glutamate; 175g = 175 μM glutamate; 250g = 250 μM glutamate. p values calculated via repeated measures ANOVA. # $p<0.05$; ###, *** $p<0.001$; ####, **** $p<0.0001$. n=5 for 0g; n=5 for 30g; n=4 for 100g; n=4 for 175g; n=4 for 250g. Grey boxes represent 95% CIs, and solid red lines represents mean. p values calculated via repeated measures ANOVA. Data from N=3 independent experiments, and n indicates number of datapoints (MEA networks). **d)** Timeline for experiments studying glutamate injury and BDNF recovery treatment.

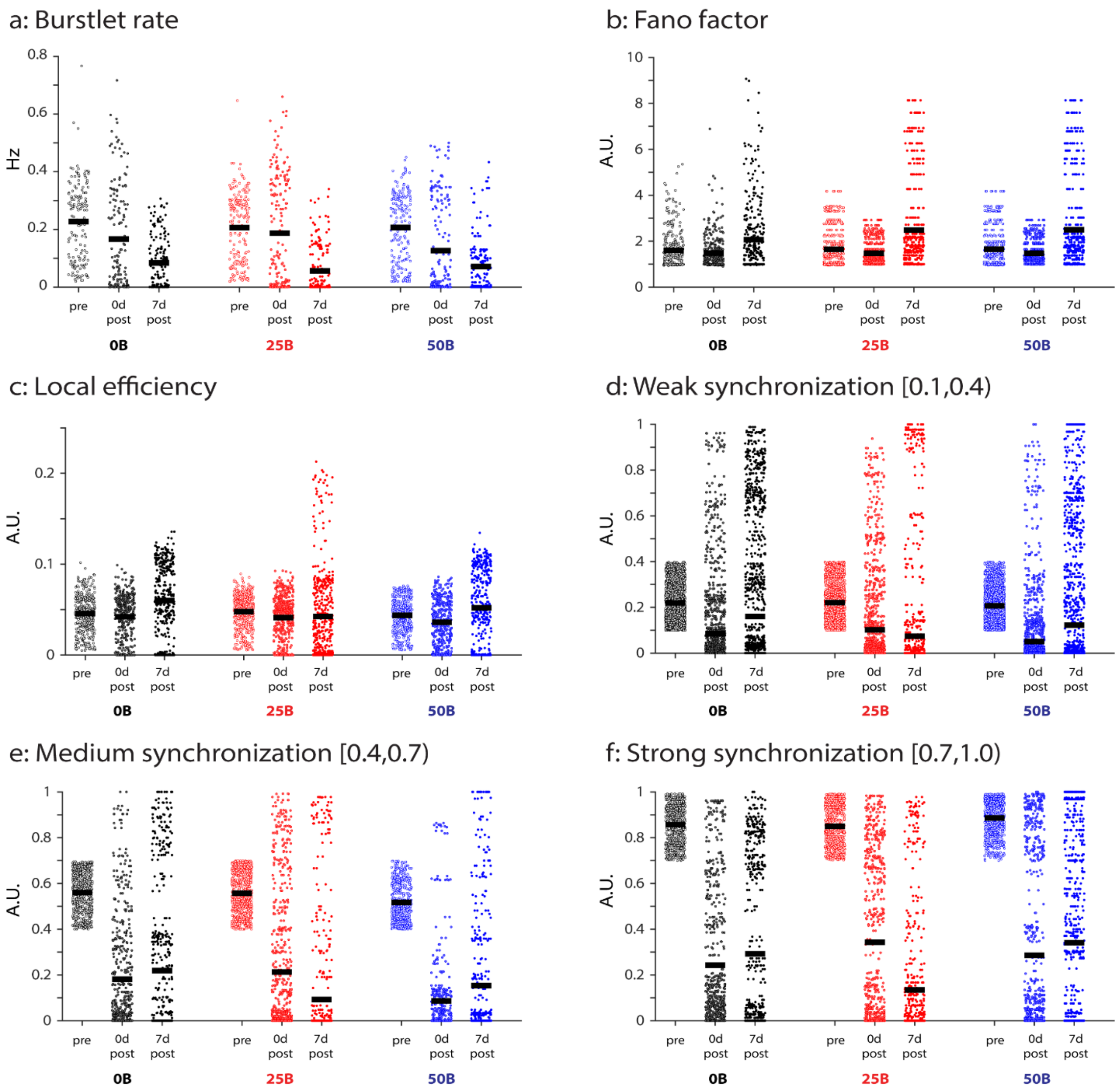

Supplementary Figure 3: Raw data demonstrating concentration-dependent effects of BDNF treatment on network dynamics. a) Changes in burstlet rate (Hz). e=158 for 0B; e=184 for 25B; e=180 for 50B. **b)** Changes in Fano factor (A.U.). e=332 for 0B; e=506 for 25B; e=452 for 50B. **c)** Changes in local efficiency (A.U.). e=356 for 0B; e=453 for 25B; e=387 for 50B. **d)** Changes in connections with initially weak synchronization (values of [0.1,0.4)). e=1720 for 0B; e=1454 for 25B; e=2002 for 50B. **e)** Changes in connections with initially medium synchronization (values of [0.4,0.7)). e=556 for 0B; e=700 for 25B; e=482 for 50B. **f)** Changes in connections with initially strong synchronization (values of [0.7,1.0)). e=620 for 0B; e=654 for 25B; e=760 for 50B. Data from N=3 independent experiments. 0B = no BDNF; 25B = 25 ng/ml BDNF; 50B = 50 ng/ml BDNF. y-axis indicates raw values. Each datapoint represents an electrode

tracked over time. Mean values are represented by solid black lines. N indicates number of experiments, and e indicates number of electrodes.

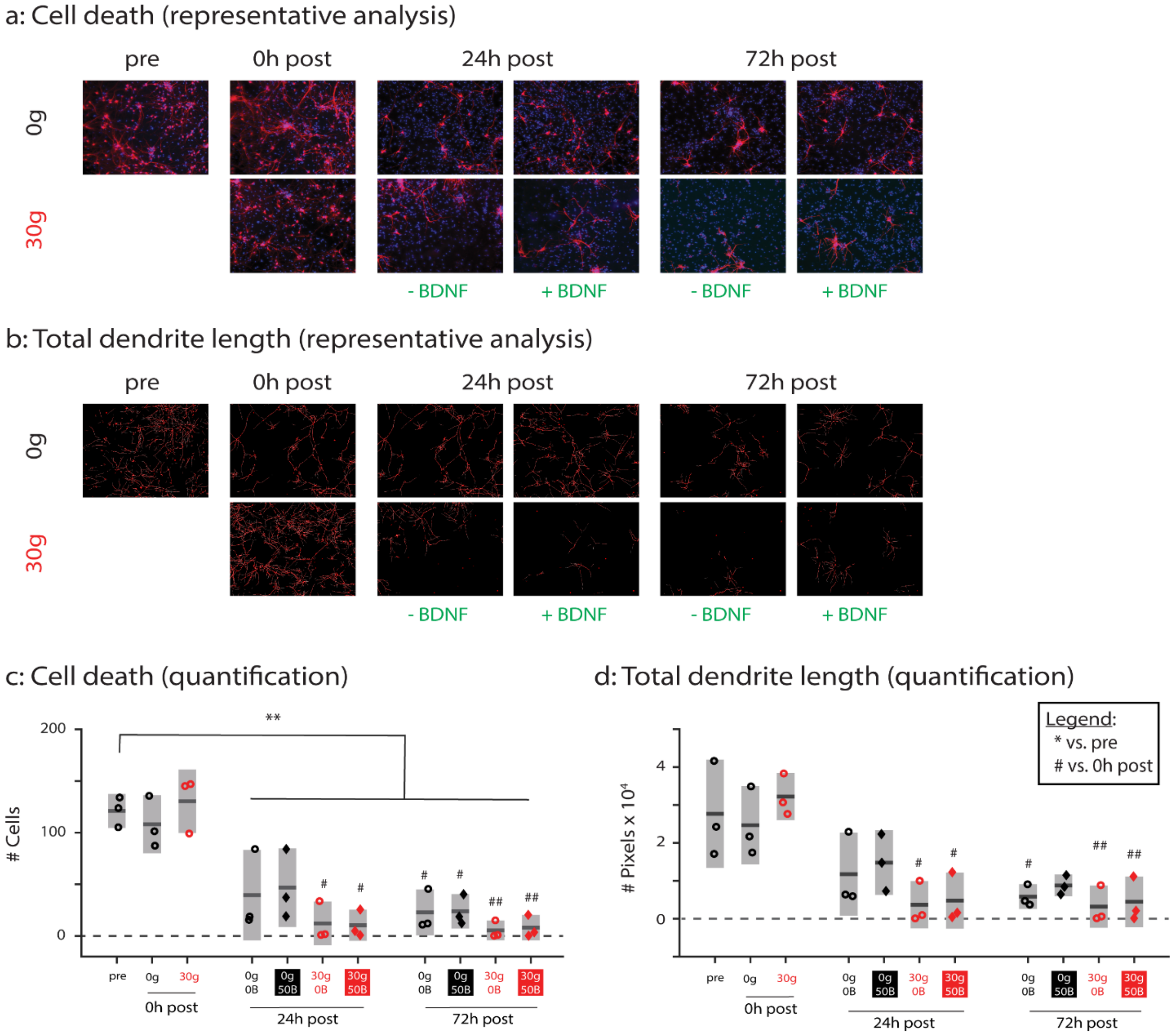

Supplementary Figure 4: BDNF does not prevent cell death or loss of dendrites after mild excitotoxic injury. a) Representative merged images of MAP2 immunostaining (red) and DAPI staining (blue) at pre-injury and 0 h, 24 h, and 72 h after injury. **b)** Representative skeletonizations of images from Fig. 5B. **c)** Quantification of number of MAP2-positive cells for each condition. BDNF does not ameliorate injury-induced cell death when used as a post-injury treatment. **d)** Quantification of total dendrite length for each condition. BDNF treatment does not ameliorate the loss in dendrite branching after injury. 0g = no glutamate; 30g = 30 µM glutamate. p values determined by RM ANOVA followed by Tukey-Kramer multiple comparisons test, where * symbols are compared to pre-treatment and # symbols are compared to 0h post (# $p<0.05$, ## $p<0.01$). Solid dark gray lines indicate mean, and gray boxes indicate 95% CIs. N=3 independent experiments with all fields of view averaged for each condition per experiment.

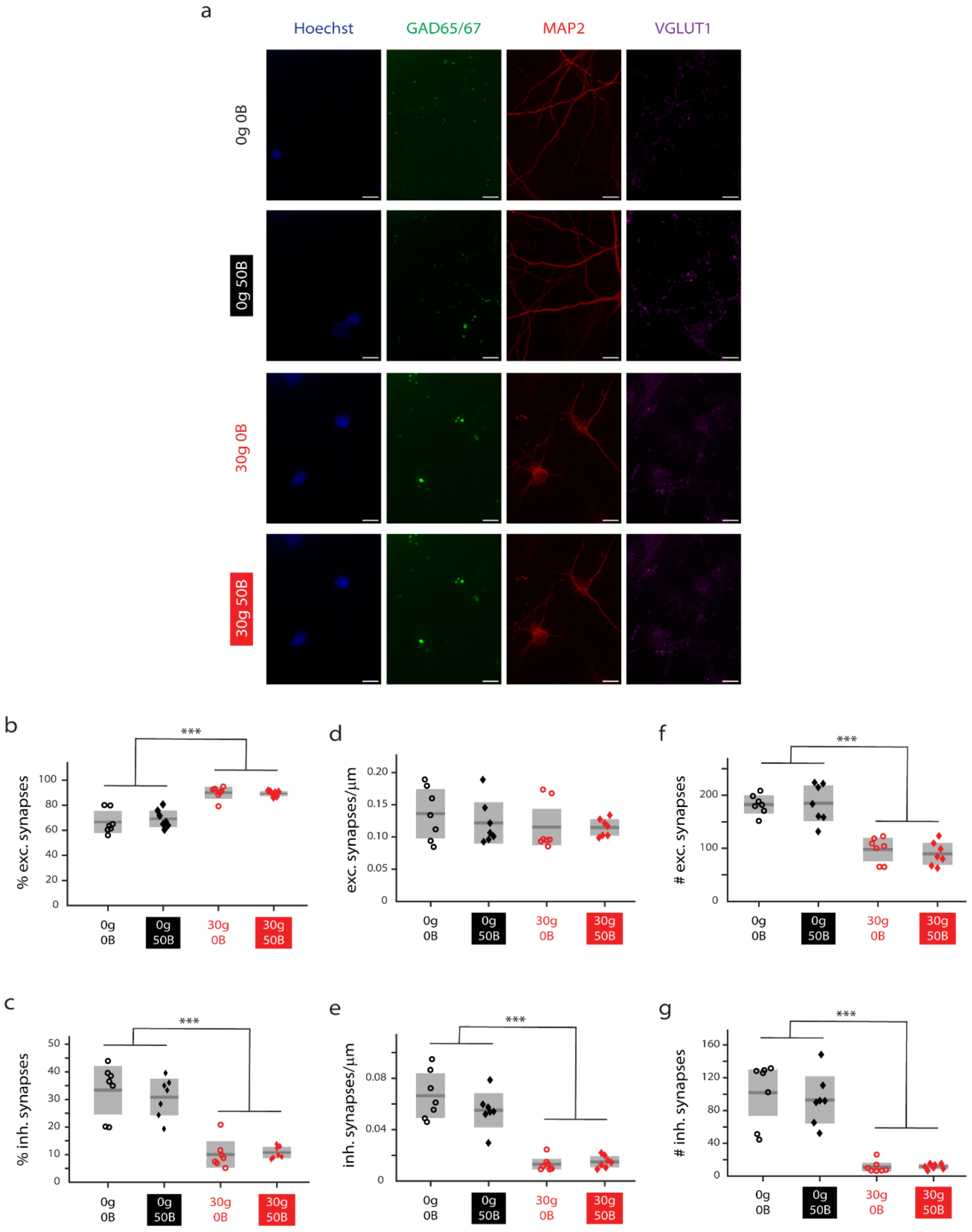

Supplementary Figure 5: Changes to excitatory and inhibitory synapse density and percentages caused by glutamate-induced excitotoxicity are not ameliorated by BDNF treatment. a) Individual channels of representative images from Fig. 5. Scale bars indicate 10 µm. **b)** The percentage of excitatory synapses significantly increases after glutamate-induced injury (both 30g 0B and 30g 50B conditions) compared to uninjured networks (both 0g 0B and 0g 50B conditions; $p<0.001$). **c)** The percentage of inhibitory synapses significantly decreases after

glutamate-induced injury (both 30g 0B and 30g 50B conditions) compared to uninjured networks (both 0g 0B and 0g 50B conditions; $p<0.001$). **d)** The density of excitatory synapses per micron of dendrite does not change with injury or BDNF treatment. **e)** The density of inhibitory synapses per micron of dendrite significantly decreases after glutamate-induced injury (both 30g 0B and 30g 50B conditions) compared to uninjured networks (both 0g 0B and 0g 50B conditions; $p<0.001$). **f)** The number of excitatory synapses significantly decreases after glutamate-induced injury (both 30g 0B and 30g 50B conditions) compared to uninjured networks (both 0g 0B and 0g 50B conditions; $p<0.001$). **g)** The number of inhibitory synapses significantly decreases after glutamate-induced injury (both 30g 0B and 30g 50B conditions) compared to uninjured networks (both 0g 0B and 0g 50B conditions; $p<0.001$). 0B = no BDNF; 50B = 50 ng/ml BDNF. p values calculated by one-way ANOVA followed by Tukey-Kramer multiple comparisons test (*** $p<0.001$), and n=7 datapoints with four fields of view averaged per coverslip. N=3 independent experiments. Gray boxes represent 95% CIs, and solid black lines represent mean. N indicates number of experiments, and n indicates number of datapoints.

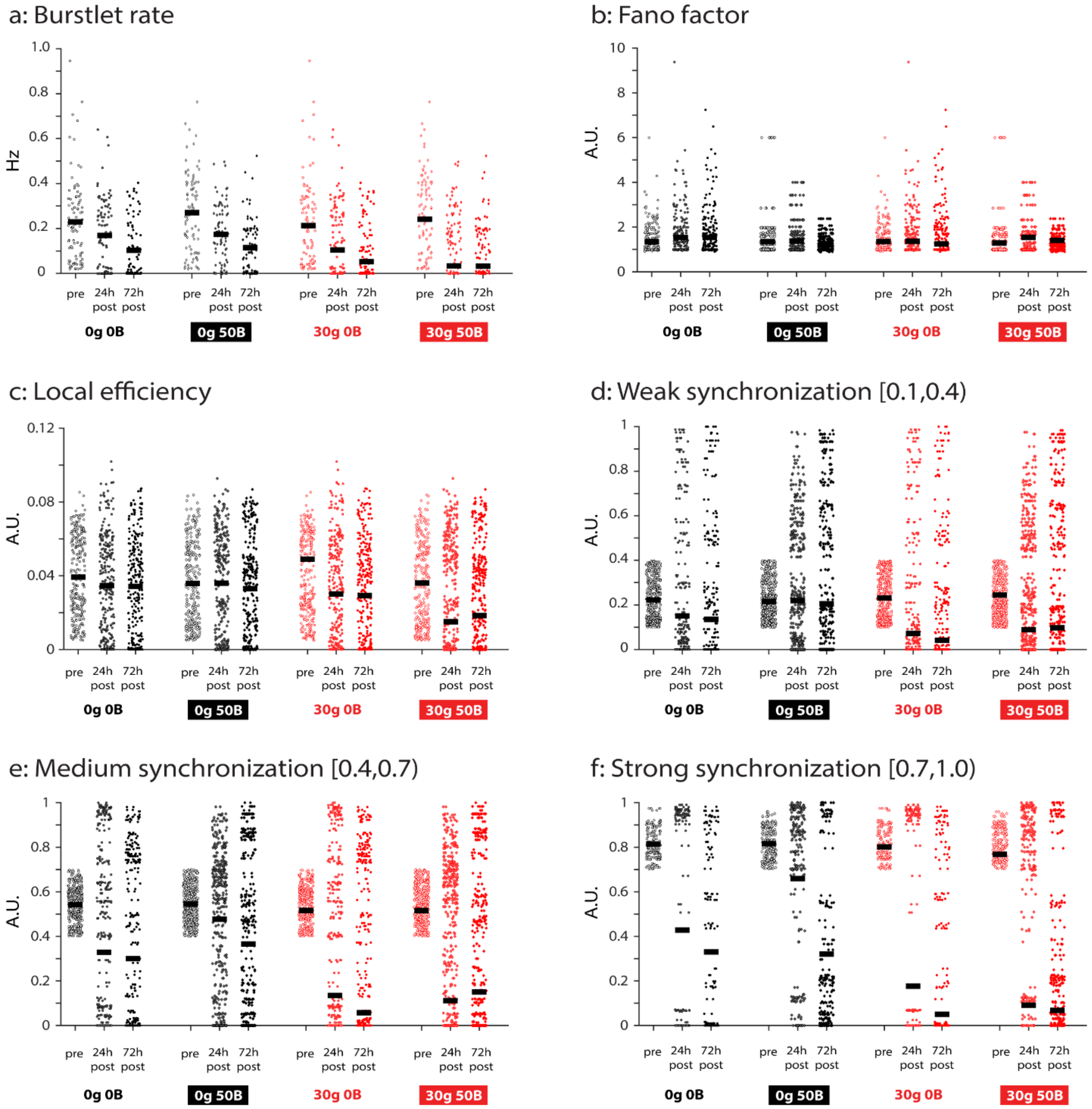

Supplementary Figure 6: Raw data demonstrating effects of excitotoxic injury and BDNF treatment on network dynamics. a) Changes in burstlet rate (Hz). e=92 for 0g0B; e=85 for 0g50B; e=91 for 30g0B; e=64 for 30g50B. **b)** Changes in Fano factor (A.U.). e=266 for 0g0B; e=315 for 0g50B; e=265 for 30g0B; e=193 for 30g50B. **c)** Changes in local efficiency (A.U.). e=268 for 0g0B; e=259 for 0g50B; e=243 for 30g0B; e=236 for 30g50B. **d)** Changes in connections with initially weak synchronization (values of [0.1,0.4)). e=412 for 0g0B; e=528 for 0g50B; e=970 for 30g0B; e=482 for 30g50B. **e)** Changes in connections with initially medium synchronization (values of [0.4,0.7)). e=366 for 0g0B; e=398 for 0g50B; e=442 for 30g0B; e=266 for 30g50B. **f)** Changes in connections with initially strong synchronization (values of [0.7,1.0)). e=170 for 0g0B; e=224 for 0g50B; e=80 for 30g0B; e=26 for 30g50B. Data from N=4 independent

experiments. 0g = no glutamate; 30g = 30 μM glutamate; 0B = no BDNF; 50B = 50 ng/ml BDNF. y-axis indicates raw values. Each datapoint represents an electrode tracked over time. Mean values are represented by solid lines. N indicates number of experiments, and e indicates number of electrodes.

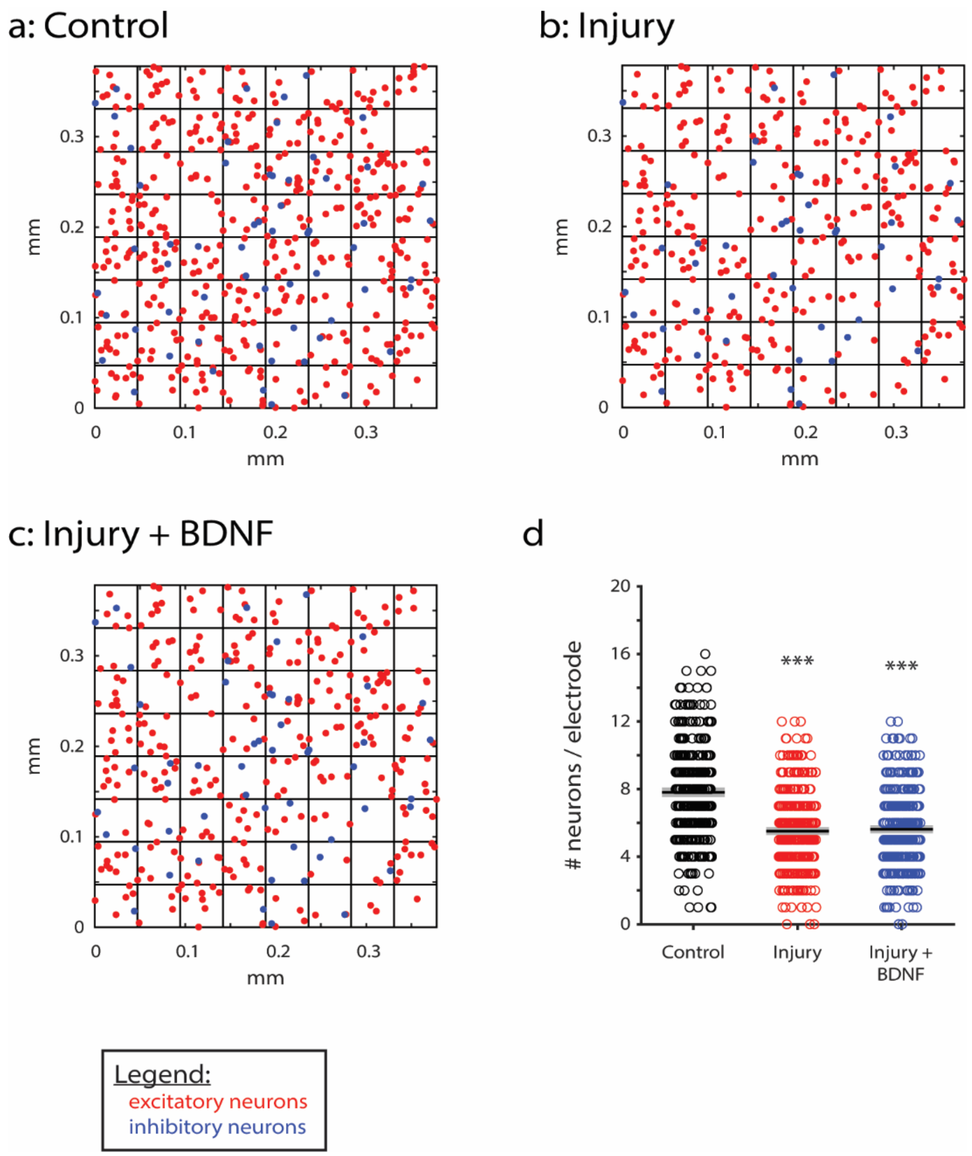

Supplementary Figure 7: Dividing *in silico* networks into MEA electrodes. a) The physical distribution of excitatory (red) and inhibitory (blue) neurons in a control network and the boundaries of the MEA regions into which the neurons would be combined. **b)** The same neuron distribution from A after injury randomly reduces the number of excitatory neurons by 30% and inhibitory neurons by 25%. **c)** The same neuron distribution from A and B after BDNF randomly reintroduces 50% of the injured inhibitory neurons. **d)** The number of neurons per electrode decreases with injury (*** $p<0.01$ via one-way ANOVA followed by Tukey-Kramer multiple comparisons test). Grey boxes represent 95% CIs, and solid lines represent mean. Each datapoint represents a simulated electrode. N=6 independent simulations, and e=384 electrodes for all conditions. For these data, we used the full 8x8 grid of 64 electrodes rather than 59 electrodes.

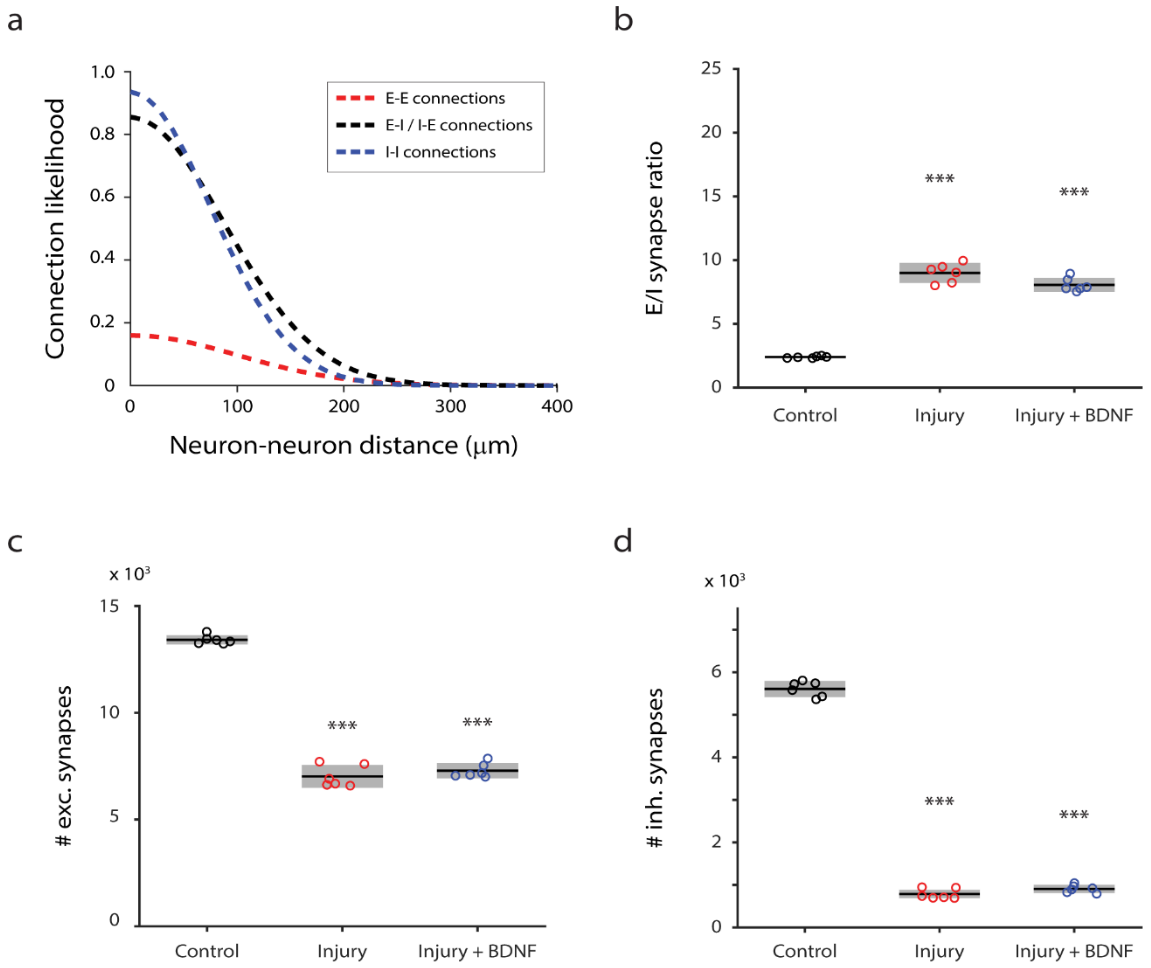

Supplementary Figure 8: Characterization of excitatory and inhibitory synapses in *in silico* model. a) Connection likelihood for e-e synapses, e-i and i-e synapses, and i-i synapses as a function of distance. **b)** E/I synapse ratio increases for Injury and Injury + BDNF compared to the Control. **c)** Number of excitatory and **d)** inhibitory synapses significantly decrease for Injury and Injury + BDNF compared to Control. *** $p<0.001$ calculated by one-way ANOVA followed by Tukey-Kramer multiple comparisons test. Gray boxes represent 95% CIs, and solid black lines represent mean. N=6 independent simulations, and each datapoint represents a simulation.

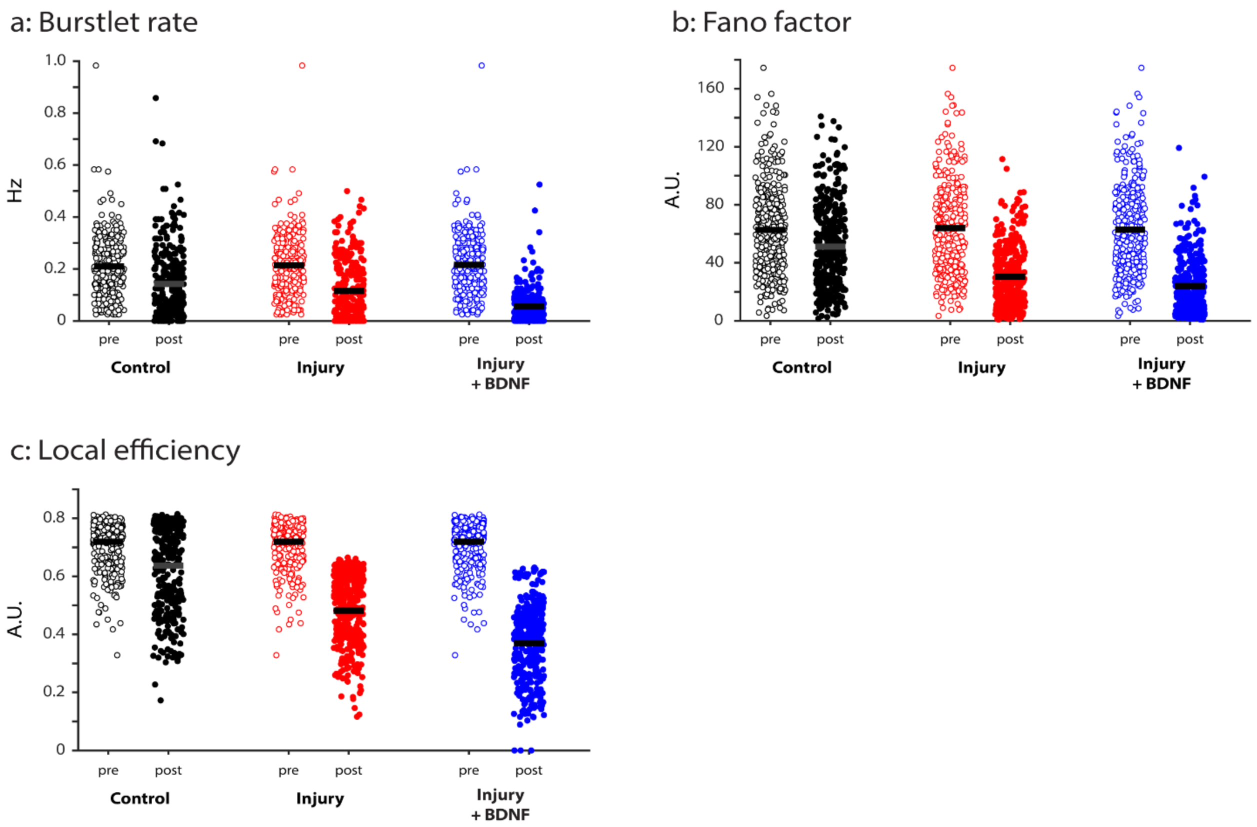

Supplementary Figure 9: Raw data demonstrating effects of injury and recovery on *in silico* networks. a) Changes in burstlet rate (Hz). e=292 for Control; e=286 for Injury; e=275 for Injury + BDNF. **b)** Changes in Fano factor (A.U.). e=354 for Control, Injury, and Injury + BDNF. **c)** Changes in local efficiency (A.U.). e=354 for Control; e=341 for Injury; e=340 for Injury + BDNF. Each datapoint represents a simulated electrode tracked over time. Mean values are represented by solid lines. N=6 independent simulations, and e represents the number of electrodes.

SUPPLEMENTARY TABLES

	**BDNF concentration** (df=2)	**Timepoint** (df=2)	**BDNF conc. & timepoint** (df=4)
**TrkB expression**	F=0.144, p=0.867	F=20.5, p<0.001	F=0.360, p=0.835
**Burstlet rate**	F=0.465, p=0.628	F=21.7, p<0.001	F=1.83, p=0.122
**Fano factor**	F=0.346, p=0.707	F=209, p<0.001	F=1.28, p=0.275
**Local efficiency**	F=10.1, p<0.001	F=47.6, p<0.001	F=14.1, p<0.001
**Weak synchronization**	F=25.7, p<0.001	F=435, p<0.001	F=40.9, p<0.001
**Medium synchronization**	F=14.4, p<0.001	F=1790, p<0.001	F=31.8, p<0.001
**Strong synchronization**	F=9.91, p<0.001	F=3210, p<0.001	F=58.7, p<0.001

Supplementary Table 1: F-statistics and p values for RM ANOVA of BDNF dose response data.

	**Treatment condition** (df=3)	**Timepoint** (df=2)	**Treatment cond. & timepoint** (df=6)
**Cell death (# cells)**	F=0.869, p=0.496	F=54.7, p<0.001	F=1.14, p=0.384
**Cell death (dendrites)**	F=0.109, p=0.953	F=28.7, p<0.001 (df=3)	F=0.811, p=0.611 (df=9)
**Burstlet rate**	F=4.19, p=0.006	F=12.2, p<0.001	F=2.51, p=0.021
**Fano factor**	F=2.00, p=0.112	F=36.9, p<0.001	F=1.83, p=0.090
**Local efficiency**	F=25.8, p<0.001	F=0.597, p=0.550	F=11.3, p<0.001
**Weak synchronization**	F=58.9, p<0.001	F=126, p<0.001	F=31.6, p<0.001
**Medium synchronization**	F=102, p<0.001	F=822, p<0.001	F=62.9, p<0.001
**Strong synchronization**	F=33.6, p<0.001	F=416, p<0.001	F=26.6, p<0.001

Supplementary Table 2: F-statistics and p values for RM ANOVA of glutamate injury and BDNF recovery data.

	**Treatment condition** (df=2)	**Timepoint** (df=1)	**Treatment cond. & timepoint** (df=2)
**Burstlet rate**	F=59.7, p<0.001	F=926, p<0.001	F=59.7, p<0.001
**Fano factor**	F=514, p<0.001	F=5480, p<0.001	F=514, p<0.001
**Local efficiency**	F=617, p<0.001	F=5210, p<0.001	F=617, p<0.001

Supplementary Table 3: F-statistics and p values for RM ANOVA of *in silico* data.
