# Supplementary material for: Time-dependent homeostatic mechanisms underlie brain-derived neurotrophic factor action on neural circuitry
Source: Commun Biol. 2023 Dec 18;6:1278. doi: 10.1038/s42003-023-05638-9 (PMC10728104; doi:10.1038/s42003-023-05638-9)
Supplement: Supplementary file 2 — Reporting summary [file 42003_2023_5638_MOESM2_ESM.pdf]

nature portfolio

Corresponding author(s): Bonnie L. Firestein

Last updated by author(s): Oct 4, 2023

Reporting Summary

Nature Portfolio wishes to improve the reproducibility of the work that we publish. This form provides structure for consistency and transparency in reporting. For further information on Nature Portfolio policies, see our Editorial Policies and the Editorial Policy Checklist.

Statistics

For all statistical analyses, confirm that the following items are present in the figure legend, table legend, main text, or Methods section.

n/a	Confirmed	
☐	☒	The exact sample size (*n*) for each experimental group/condition, given as a discrete number and unit of measurement
☐	☒	A statement on whether measurements were taken from distinct samples or whether the same sample was measured repeatedly
☐	☒	The statistical test(s) used AND whether they are one- or two-sided<br>*Only common tests should be described solely by name; describe more complex techniques in the Methods section.*
☒	☐	A description of all covariates tested
☐	☒	A description of any assumptions or corrections, such as tests of normality and adjustment for multiple comparisons
☐	☒	A full description of the statistical parameters including central tendency (e.g. means) or other basic estimates (e.g. regression coefficient) AND variation (e.g. standard deviation) or associated estimates of uncertainty (e.g. confidence intervals)
☐	☒	For null hypothesis testing, the test statistic (e.g. *F*, *t*, *r*) with confidence intervals, effect sizes, degrees of freedom and *P* value noted<br>*Give P values as exact values whenever suitable.*
☒	☐	For Bayesian analysis, information on the choice of priors and Markov chain Monte Carlo settings
☒	☐	For hierarchical and complex designs, identification of the appropriate level for tests and full reporting of outcomes
☒	☐	Estimates of effect sizes (e.g. Cohen's *d*, Pearson's *r*), indicating how they were calculated

Our web collection on statistics for biologists contains articles on many of the points above.

Software and code

Policy information about availability of computer code

Data collection

The data acquisition software used was MC_Rack (Multi Channel Systems, Germany, version 4.6.2).

Data analysis

All code will be made available on GitHub and ModelDB. As described in the manuscript, we used MATLAB 2021a (Mathworks) for all data analysis, data plotting, and statistical tests with the exception of in silico simulations (run in Python 3.8).

Extracting parameters from MEA recording data: As in our previous work [Kutzing et al 2011, DOI: 10.1007/s10439-011-0319-0; Kutzing et al 2012, DOI: 10.1007/s10439-011-0494-z; Rodriguez et al 2018, DOI: 10.1088/1741-2552/aa976a; Rodriguez et al 2021, DOI: 10.1162/netn_a_00173], we used MEAtools [U Egert et al 2002, DOI: 10.1016/S0165-0270(02)00045-6] to extract raw spiking data from *.mcd recording files. We detected spikes and calculated downstream parameters as in recent publications [Rodriguez et al 2018, DOI: 10.1088/1741-2552/aa976a; Rodriguez et al 2021, DOI: 10.1162/netn_a_00173], which is described in detail in our manuscript.

Extracting dendrites and synapses from immunostaining data: We use a previously published image analysis method (Laplacian of Gaussian filtering) to identify dendrites in fluorescence images [Campanello et al 2021, DOI: 10.1371/journal.pcbi.1007986; O'Neill et al 2022, DOI: 10.1101/2021.09.13.460152]. We used size thresholding along with proximity to dendrites to identify synapses.

Granger causal analysis: We use Granger causal analysis that we and others have published [Francis et al 2018, DOI: 10.1016/j.neuron.2018.01.019; Francis et al 2022, DOI: 10.1016/j.celrep.2022.110878; Bressler & Seth 2011, DOI: 10.1016/j.neuroimage.2010.02.059; Ding et al 2006, DOI: 10.1002/9783527609970.CH17; Seth et al 2015, DOI: 10.1523/JNEUROSCI.4399-14.2015] and extend it to spiking data [Kim et al 2011, DOI: 10.1371/journal.pcbi.1001110; Sheikhattar et al 2018, DOI: 10.1073/pnas.1718154115].

In silico simulations: In this work, we use the published Brian2 neural simulator [Masquelier & Deco 2013, DOI: 10.1371/

journal.pone.0075824; Stimberg et al 2019, DOI: 10.7554/eLife.47314] and adapted the simulation paradigm to in vitro data as we have done previously [Rodriguez et al 2021, DOI: 10.1162/netn_a_00173].

For manuscripts utilizing custom algorithms or software that are central to the research but not yet described in published literature, software must be made available to editors and reviewers. We strongly encourage code deposition in a community repository (e.g. GitHub). See the Nature Portfolio guidelines for submitting code & software for further information.

Data

Policy information about availability of data

All manuscripts must include a data availability statement. This statement should provide the following information, where applicable:

- Accession codes, unique identifiers, or web links for publicly available datasets
- A description of any restrictions on data availability
- For clinical datasets or third party data, please ensure that the statement adheres to our policy

All data will be made available on GitHub.

Human research participants

Policy information about studies involving human research participants and Sex and Gender in Research.

Reporting on sex and gender	N/A
Population characteristics	N/A
Recruitment	N/A
Ethics oversight	N/A

Note that full information on the approval of the study protocol must also be provided in the manuscript.

Field-specific reporting

Please select the one below that is the best fit for your research. If you are not sure, read the appropriate sections before making your selection.

☒ Life sciences ☐ Behavioural & social sciences ☐ Ecological, evolutionary & environmental sciences

For a reference copy of the document with all sections, see nature.com/documents/nr-reporting-summary-flat.pdf

Life sciences study design

All studies must disclose on these points even when the disclosure is negative.

Sample size

Sample size was not predetermined. We used the following rationales and our previous experiments, with n values reported in our published papers:

MEA recording experiments: We aimed for at least three independent experiments with multiple MEAs (cultures) per condition as in our previous work [Kutzing et al 2011, DOI: 10.1007/s10439-011-0319-0; Kutzing et al 2012, DOI: 10.1007/s10439-011-0494-z; Rodriguez et al 2018, DOI: 10.1088/1741-2552/aa976a; Rodriguez et al 2021, DOI: 10.1162/netn_a_00173]. Unfortunately, repeating these experiments was largely limited by feasibility because microelectrode arrays have a limited lifetime of use. However, an advantage of microelectrode arrays is being able to calculate parameters for each electrode and track each electrode over time.

Western blot analysis: We performed seven independent experiments, which is similar to or with n value greater than what we have reported in the past [Kwon et al 2011, DOI: 10.1523/JNEUROSCI.6785-10.2011; Patel et al 2018, DOI: 10.1007/s12035-017-0849-z; Rodriguez et al 2021, DOI: 10.1162/netn_a_00173].

Cell death and excitatory/inhibitory experiments: We performed three separate experiments. We averaged multiple fields of view from multiple wells for each datapoint, similar to what we previously reported [Du et al 2007, DOI: 10.1002/glia.20472].

In silico experiments: We performed six independent simulations in line with our previous work [Rodriguez et al 2021, DOI: 10.1162/netn_a_00173].

Data exclusions

For MEA recording experiments in general: As stated in the manuscript, cultures with less than 2000 spikes in 5 min (a spike rate of less than 6.7 Hz for the whole culture) were not used for further experimentation. This ensured that the cultures were active enough for positive or negative changes to be observed. This criterion was pre-established.

For MEA data where electrodes are datapoints: We used percent change to represent how activity of individual electrodes change as a result of treatment. As stated in the manuscript, to prevent artificial inflation of percent change values, the following thresholds (per electrode) were used: 0.2 Hz for spike rate, 0.02 Hz for burstlet rate, 0.01 Hz for global burst rate, and 0.005 (A.U.) for local efficiency. Any electrodes not

	meeting these thresholds were not used for analysis. This criterion was pre-established prior to performing statistical tests.<br><br>For MEA data where cultures are datapoints: in general, no exclusions. The exception is in the data in Suppl. Fig. 2C. Outlier elimination was based on median absolute deviation (MAD) calculation (eliminate outliers more than two scaled MAD away from the median). This eliminated two datapoints from the control condition. This is a pre-established method used by our lab for outlier elimination.<br><br>For Western blot analysis: Outlier elimination was based on median absolute deviation (MAD) calculation (eliminate outliers more than two scaled MAD away from the median). We used the normalized data to detect outliers. This eliminated one experiment (set #3). This is a pre-established method used by our lab for outlier elimination.<br><br>For cell death and excitatory-inhibitory neuron/synaptic analysis: no exclusions.<br><br>For in silico data: We eliminated outliers from the final (normalized) data using the MAD calculation described above.
Replication	To ensure replication of MEA data, we implemented a minimum activity threshold. As stated in the manuscript, cultures with less than 2000 spikes in 5 min (a spike rate of less than 6.7 Hz for the whole culture) were not used for further experimentation. This ensured that the cultures were active enough for positive or negative changes to be observed.<br><br>To ensure replication of Western blot analysis and cell death experiments, all dissections, culturing, and experiments were performed by the same person and with the same antibodies in as short a time as feasible.
Randomization	As stated in the manuscript, cultures were randomly assigned conditions for MEA recordings, Western blot analysis, and cell death imaging. Covariates are not relevant.
Blinding	We used blinding when imaging the cell death experiments to ensure that the fields of view captured for analysis were truly random and not biased. For the rest of our data analysis, blinding is not relevant since all data analysis was automated, and the same parameters/thresholds were pre-determined and used regardless of condition.

Reporting for specific materials, systems and methods

We require information from authors about some types of materials, experimental systems and methods used in many studies. Here, indicate whether each material, system or method listed is relevant to your study. If you are not sure if a list item applies to your research, read the appropriate section before selecting a response.

Materials & experimental systems

n/a	Involved in the study
☐	☒ Antibodies
☒	☐ Eukaryotic cell lines
☒	☐ Palaeontology and archaeology
☐	☒ Animals and other organisms
☒	☐ Clinical data
☒	☐ Dual use research of concern

Methods

n/a	Involved in the study
☒	☐ ChIP-seq
☒	☐ Flow cytometry
☒	☐ MRI-based neuroimaging

Antibodies

Antibodies used	Antibodies used for Western blot analysis: rabbit anti-TrkB antibody as the primary antibody (Cell Signalling Technology; cat. no. 4603T; clone 80E3) and goat anti-rabbit HRP-conjugated antibody as the secondary antibody (Rockland Immunochemicals, Inc.; cat. no. 611-1302).<br><br>Antibodies used for cell death immunostaining: mouse anti-MAP2 as the primary antibody (BD Biosciences; cat. no. 556320; clone Ap20), donkey anti-mouse AlexaFluor 488 as the secondary antibody (ThermoFisher; cat. no. A32766), and Hoechst 33342 to mark nuclei (Millipore Sigma; cat. no. B2261).<br><br>Antibodies used for assessment of excitatory-inhibitory neuronal and synaptic balance. Primary antibodies: polyclonal chicken anti-MAP2 (1:1000; Novus Biologicals, cat. no. NB300-213), mouse anti-VGLUT1 (1:250; Synaptic Systems, cat. no. 135 011, clone 68B7), rabbit anti-GAD65/67 (1:250; Abcam, cat. no. ab183999, clone EPR19366). Secondary antibodies: goat anti-rabbit IgG AlexaFluor 488 (1:1000; ThermoFisher/Invitrogen, cat. no. A-11008), goat anti-chicken IgY AlexaFluor 555 (1:1000; ThermoFisher/Invitrogen, cat. no. A-21437), donkey anti-mouse IgG AlexaFluor 647 (1:1000; ThermoFisher/Invitrogen, cat. no. A-31571).
Validation	Each primary antibody used in this work has been validated by multiple other publications (including 4 examples for each antibody):<br><br>rabbit anti-TrkB antibody from Cell Signalling Technology (cat. no. 4603T, clone 80E3) -- publications also using this antibody for Western blot analysis with rat brain cells/tissue include Chen et al 2012 (DOI: 10.1038/nn.3266); Travaglia et al 2016 (DOI: 10.1038/nn.4348), Xe et al 2017 (DOI: 10.1038/nn.4443), and Tolo et al 2018 (DOI: 10.3389/fnmol.2018.00049).<br><br>mouse anti-MAP2 antibody from BD Biosciences (cat. no. 556320, clone Ap20) -- publications also using this antibody for immunocytochemistry in rat brain cells include O'Neill et al 2015 (DOI: 10.3389/fncel.2015.00285), O'Neill et al 2018 (DOI: 10.3389/fncel.2018.00060), Rodriguez et al 2018 (DOI: 10.1088/1741-2552/aa976a), and Patel et al 2019 (DOI: 10.1089/neu.2018.6291).

polyclonal chicken anti-MAP2 antibody from Novus Biologicals (cat. no. NB300-213) -- publications also using this antibody for immunocytochemistry in rat brain cells include Falkovich et al 2023 (DOI: 10.1016/j.celrep.2023.112430), Bulumulla et al 2022 (DOI: 10.7554/eLife.78773), Jablonski et al 2021 (DOI: 10.1038/s41598-021-82901-1), and Hyvärinen et al 2019 (DOI: 10.1038/s41598-019-53647-8).

mouse anti-VGLUT1 from Synaptic Systems (cat. no. 135 011, clone 68B7) -- publications also using this antibody for immunocytochemistry in rodent brain cells include Hyun et al 2022 (DOI: 10.1242/dmm.049177), Klatt et al 2021 (DOI: 10.1016/j.celrep.2021.109266), McCabe et al 2021 (DOI: 10.1523/ENEURO.0269-20.2020), and Moore et al 2019 (DOI: 10.1038/s41398-018-0344-y).

rabbit anti-GAD65/67 from Abcam (cat. no. ab183999, clone EPR19366) -- publications also using this antibody for immunocytochemistry in rodent brain cells include Miranda et al 2022 (DOI: 10.1002/cne.25232), Potier et al 2022 (DOI: 10.3390/ijms23020592), Hu et al 2022 (DOI: 10.1186/s12915-022-01405-0), and Lee et al 2019 (DOI: 10.1038/s41586-019-1053-2).

Animals and other research organisms

Policy information about studies involving animals; ARRIVE guidelines recommended for reporting animal research, and Sex and Gender in Research

Laboratory animals	In this work, we isolated primary hippocampal neurons from euthanized Sprague Dawley embryonic rats, purchased from Taconic.
Wild animals	N/A
Reporting on sex	We combined cells from embryos of both sexes and therefore did not differentiate between sex.
Field-collected samples	N/A
Ethics oversight	The following statement is included in the manuscript: All studies involving animals were performed in accordance with and approved by the Institutional Animal Care and Use Committee (IACUC) at Rutgers University.

Note that full information on the approval of the study protocol must also be provided in the manuscript.
